# GZMK-expressing tissue-resident memory CD8^+^ T cells participate in human intestinal acute graft-versus-host disease

**DOI:** 10.1172/jci.insight.199156

**Published:** 2026-07-22

**Authors:** Yiming Sun, Yutong Xue, Chenyuyao Song, Xinyu Liu, Ruoyang Shao, Zhiping Fan, Ren Lin, Fen Huang, Na Xu, Li Xuan, Min Dai, Jing Sun, Qifa Liu, Hua Jin

**Affiliations:** 1 Department of Hematology, Nanfang Hospital, Southern Medical University, Guangzhou, China.; 2 Clinical Medical Research Center of Hematological Diseases of Guangdong Province, Guangzhou, China.

**Keywords:** Autoimmunity, Hematology, Immunology, Bone marrow transplantation, T cells

## Abstract

Intestinal acute graft-versus-host disease (aGVHD) is a common life-threatening complication of allogeneic hematopoietic stem cell transplantation (allo-HSCT). Although tissue-resident memory T (TRM) cells are thought to play a pathophysiological role in animal models of aGVHD, little is known about the role of distinct subsets of TRM cells in human intestinal aGVHD. Herein, we combined multiplex immunohistochemical staining with single-cell RNA sequencing to elucidate the differentiation trajectory, lineage commitment, clonal expansion, and functional properties of distinct CD8^+^ TRM cell subsets in human intestinal aGVHD. We identified a predominant GZMK^+^CD8^+^ TRM subset, characterized by the GZMK and CD49A markers. Intestinal aGVHD was associated with infiltration of GZMK^+^CD8^+^ T cells with TRM features, which showed enhanced clonal expansion, IFN signaling pathway–associated proinflammatory pathway expression, and lineage bifurcation differentiation properties. High GZMK^+^CD8^+^ TRM subset infiltration was associated with greater human intestinal aGVHD severity and poor prognosis. Together, our studies highlight the importance of the GZMK^+^CD8^+^ TRM subset in human intestinal aGVHD, and interest for designing GZMK^+^CD8^+^ TRM cell–targeted therapies.

## Abstract 2

Researchers identified a GZMK-expressing CD8+T cell subset linked to severe intestinal GVHD after stem cell transplantation and poorer patient outcomes.

## Graphical abstract

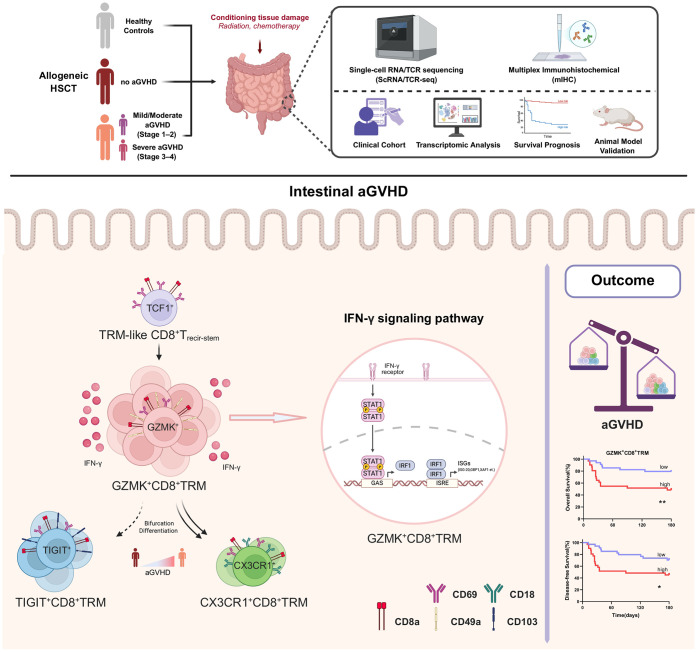

## Introduction

Allogeneic hematopoietic stem cell transplantation (allo-HSCT) is an effective therapeutic strategy for a variety of disorders, including hematological malignancies and immune deficiencies (1). Nevertheless, graft-versus-host disease (GVHD) remains a frequent and potentially life-threatening complication after allo-HSCT, and continues to be a major barrier to successful transplantation outcomes (2).

Most current knowledge regarding human GVHD after allo-HSCT has been derived from studies of circulating immune cells. However, peripheral blood captures only part of the immune response, whereas many immune cells are located in non-lymphoid tissues. Among these tissue-localized immune populations, tissue-resident memory T (TRM) cells can persist within peripheral organs for prolonged periods (3, 4). TRM cells are particularly common in border tissues such as the intestine (5), lung (6, 7), and skin (8), which are also major target tissues of GVHD after allo-HSCT.

TRM cells were first characterized in mouse tissues through experimental approaches designed to distinguish tissue-retained T cells from circulating T cells (9–11). CD69 and CD103 have been widely used as markers to identify TRM cells, although human TRM cells may display more heterogeneous phenotypes depending on tissue context and disease state (12–14). In mouse models, TRM cells reportedly mediate rapid protective immunity (15, 16) and contribute to the propagation of autoinflammatory diseases (12, 17). Studies of human TRM cells have often been guided by findings from mouse models, including the use of CD69 as a surrogate marker for human TRM cells (18, 19). Moreover, several elegant studies have previously attempted to track the long-term maintenance of bona fide human TRM cells. Bartolome et al. reported that TRM cells persist in organ transplant patients for more than 1 year (20). Snyder et al. showed that CD4^+^ and CD8^+^ TRM cells are detectable in the lung and that their persistence is associated with clinical outcomes after lung transplantation (21). Recently, the role of TRM cells in human tissue-specific immune and inflammatory diseases has been recognized (22). Given the emerging importance of TRM cells, the assessment of the immune regulation of human TRM cells in GVHD is essential.

Sherrie et al. demonstrated that host skin-resident T cells can be activated by donor monocytes and contribute to GVHD-like dermatitis in a humanized mouse model (23). In a rhesus macaque allo-HSCT model, Victor et al. used serial intravascular staining combined with single-cell RNA sequencing (scRNA-seq) to demonstrate that donor CD8^+^ T cells with invasive and tissue-resident features are involved in gastrointestinal acute GVHD (aGVHD) (24). However, studies in animal models may not accurately reflect what is observed in human tissues. In humans, Georg et al. demonstrated that long-term persisting TRM cells of host origin in the skin participate in skin GVHD pathogenesis (25). Zielinski et al. reported that the long-term persistence of human host skin TRM cells maintain recirculation potential, but is not correlated with the development of chronic GVHD (3). However, the composition, differentiation states, and clinical relevance of intestinal CD8^+^ TRM subsets in human aGVHD remain incompletely defined.

Herein, we identified a GZMK-expressing CD8^+^ TRM subset in human intestinal aGVHD tissues by combining multiplex immunohistochemical (mIHC) staining with scRNA-seq. GZMK-expressing CD8^+^ TRM cells, as the predominant CD8^+^ TRM subset, exhibited expanded clonality, IFN signaling pathway–associated proinflammatory signatures, and lineage bifurcation differentiation properties. Overall, our findings show that human intestinal aGVHD is associated with infiltration of a GZMK-expressing CD8^+^ TRM subset.

## Results

### Donor-derived CD8^+^ TRM cells are enriched in the intestines of patients with aGVHD.

**Figure 1:**
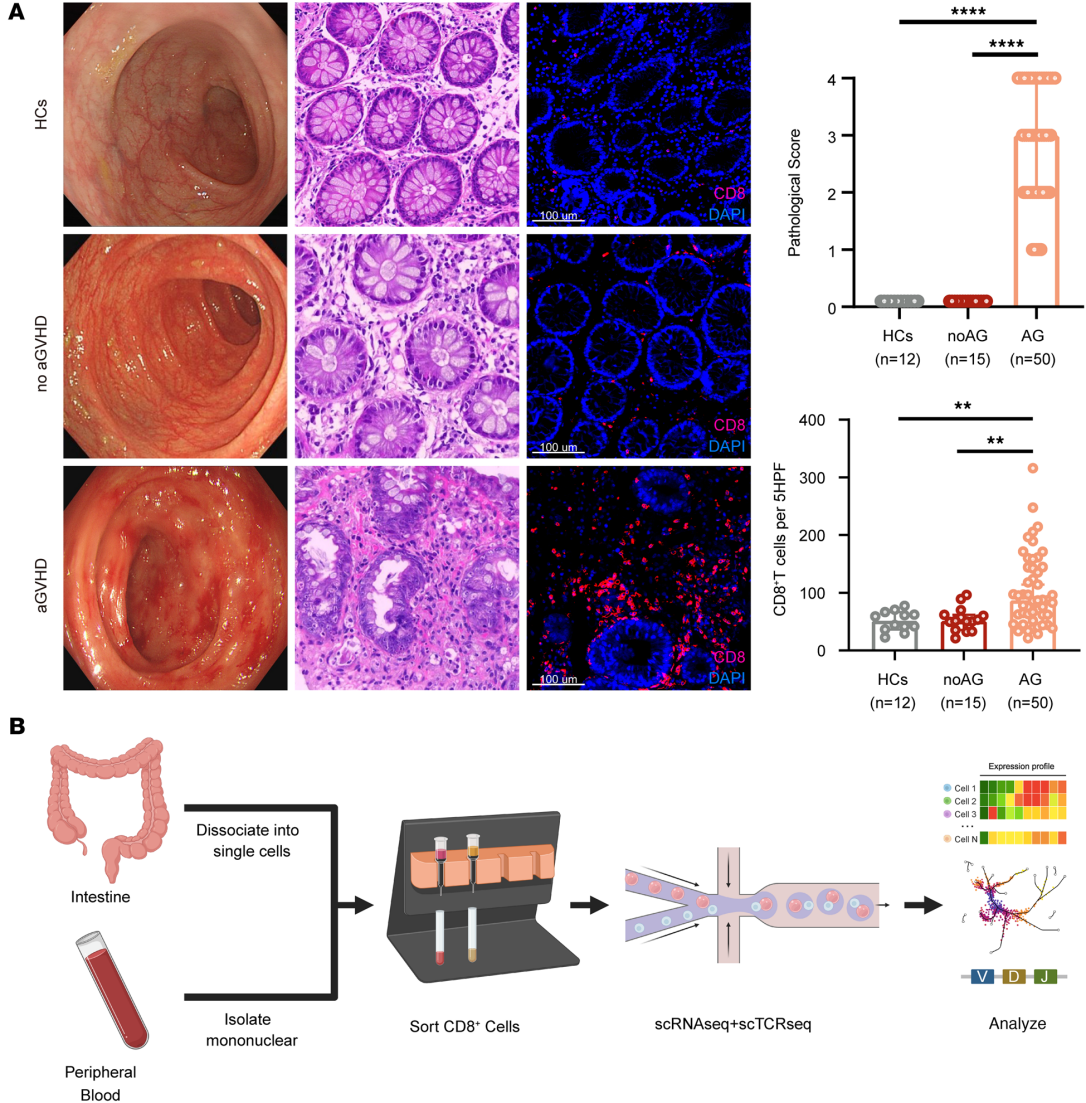
CD8^+^ T cells are enriched in the intestines of patients with aGVHD. (**A**) Representative endoscopic images, histopathology staining, and immunostaining of CD8^+^ T cells (left) and histologic grading and CD8^+^ T cell quantification (right) in patients who received allo-HSCT with intestinal aGVHD (*n* = 12), without aGVHD (*n* = 15), and healthy controls (HCs) (*n* = 50). HPF, high-power field (×40). **P < 0.01; ****P < 0.0001 according to the Wilcoxon test. Scale bars: 100 μm. (**B**) CD8^+^ T cells were sorted from the intestinal tissues and peripheral blood (PB) of aGVHD patients for scRNA-seq and scTCR-seq analyses.

**Figure 2:**
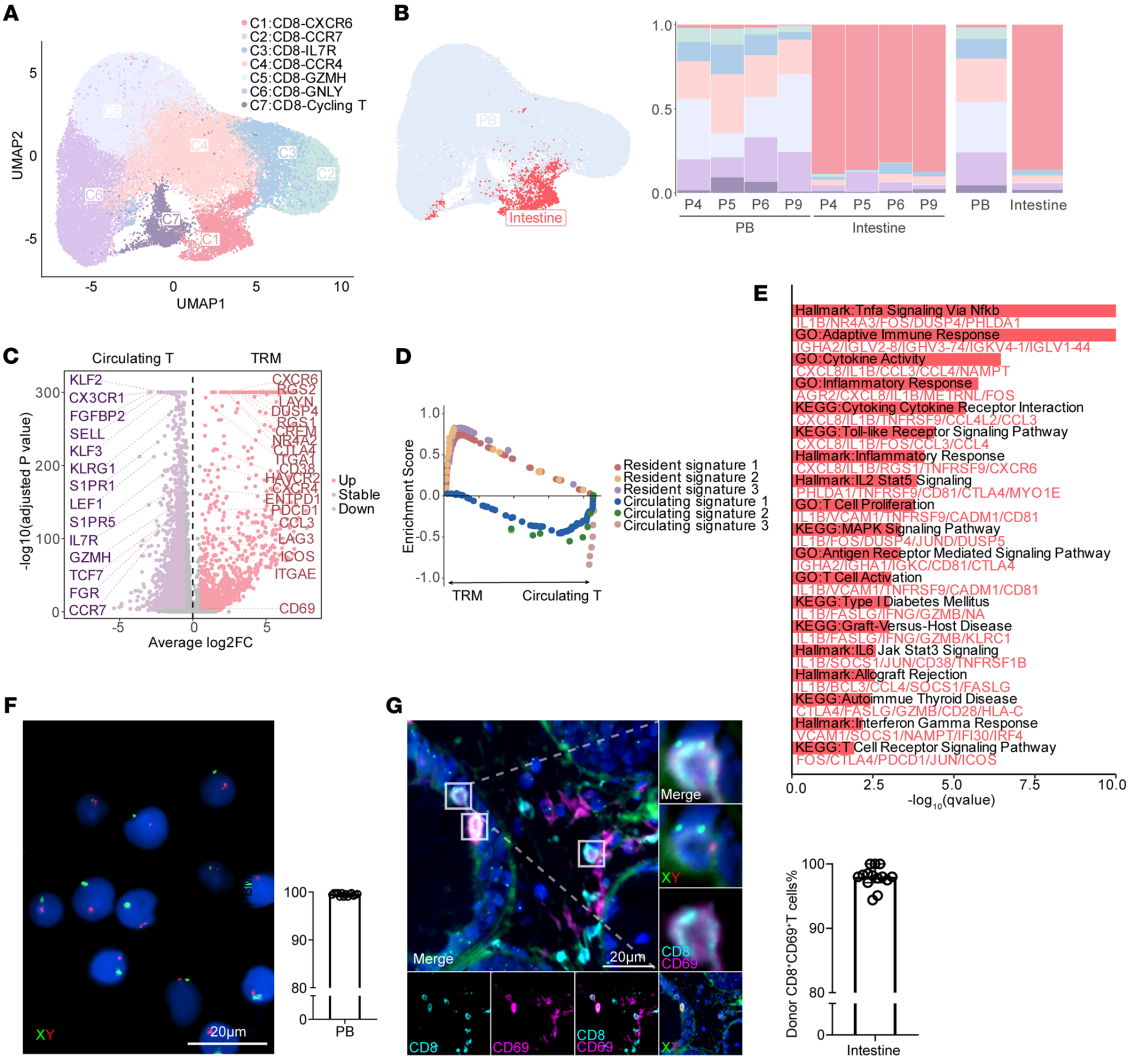
Donor-derived CD8^+^ TRM cells are enriched in the intestines of aGVHD patients. (**A**) Uniform manifold approximation and projection (UMAP) plot of 46,346 CD8^+^ T cells from 4 intestinal aGVHD patients, which demonstrated subclustering of CD8^+^ T cells into 7 phenotypes. Each dot represents a single cell (left). (**B**) Left: UMAP plot of the distribution of CD8^+^ T cell clusters separated according to intestinal tissues and peripheral blood (PB). Right: Stacked bar plots of the distribution of CD8^+^ T cell clusters in each sample. (**C**) Volcano plots showing the differentially expressed genes (DEGs) between CD8^+^ resident memory T (TRM) cells and CD8^+^ circulating T cells. Benjamini-Hochberg–adjusted *P* values ≤ 0.05 and log_2_(fold change) (log_2_FC) values ≥ 0.25 were calculated via the Wilcoxon rank-sum test. Respective genes were annotated. (**D**) Gene set enrichment analysis (GSEA) curves for resident and circulating signatures in CD8^+^ TRM cells and CD8^+^ circulating T cells. (**E**) GSEA (KEGG, HALLMARK, and GO) analyses were performed on highly expressed genes in CD8^+^ TRM cells. (**F**) Left: Representative image of FISH for X (green) and Y (red) chromosomes in the peripheral blood mononuclear cells (PBMCs) of an allo-HSCT patient (female recipient received male allograft). Right: Proportion of donor cells in PBMCs (*n* = 14). (**G**) Representative image of combined immunostaining and FISH-XY in the intestine of an allo-HSCT patient (female recipient received male allograft). Left: The white boxes indicate donor CD8^+^CD69^+^ TRM cells. Right: Proportion of donor TRM cells in intestinal TRM cells (*n* = 14). Scale bars: 20 μm (**F** and **G**).

To gain insight into the mechanisms of the pathophysiology of intestinal aGVHD, we analyzed intestinal biopsy samples from 65 patients who received allo-HSCT (50 patients with intestinal aGVHD and 15 patients without intestinal aGVHD) and 12 untransplanted healthy controls (HCs) (26). Detailed clinical and pathological information are provided in Supplemental Table 1 (supplemental material available online with this article; https://doi.org/10.1172/jci.insight.199156DS1). Baseline patient characteristics did not significantly differ between the groups with and without intestinal aGVHD (Table 1, columns 2 and 3). Compared with those in untransplanted HCs and patients without intestinal aGVHD, endoscopic findings in patients with intestinal aGVHD demonstrated mucosal inflammation, including edema, erythema, erosions, superficial ulcerations, sloughing, and denudation. aGVHD intestines also exhibited the typical histopathology of aGVHD, including lymphocytic infiltration, epithelial cell apoptosis, and crypt dropout, and significantly increased CD8^+^ T cell infiltration (*P* < 0.0001 and *P* < 0.01; Figure 1A). We isolated CD8^+^ T cells from the intestinal tissues of 4 patients with intestinal aGVHD and matched peripheral blood for whole-transcriptome and VDJ profiling via scRNA-seq, after which we paired single-cell T cell receptor sequencing (scTCR-seq) to obtain the basic properties of the CD8^+^ T cell subsets (27) (Figure 1B). The distributions of the CD8^+^ T cell clusters within the peripheral blood and intestinal tissue differed; specifically, the C1:CD8^+^ TRM cluster was almost exclusively located in the intestinal tissue (28, 29), whereas the other clusters were prevalent in the peripheral blood (Supplemental Figure 1). Accordingly, the CD8^+^ TRM cluster exhibited significant enrichment of the tissue residency signature, along with a decreased core circulating signature, compared with the other clusters (11) (*P* < 0.0001; Figure 2, A and B, and Supplemental Figure 1D). To further determine the heterogeneity of the CD8^+^ TRM cluster compared with the other clusters, we identified 5,619 differentially expressed genes (DEGs), including 367 upregulated genes and 5,252 downregulated genes (Supplemental Table 2). Compared with the other clusters, CD8^+^ TRM clusters in the intestine demonstrated downregulated *S1PR1* (encoding a *CD69* antagonist) and its transcription factor *KLF2*, the related Kruppel-like transcription factor *KLF3*, the chemokine receptor *CX3CR1*, and the lymph node–homing molecules *SELL* (CD62L) and *CCR7*, all of which control T cell egress, migration and tissue retention (30). Conversely, in addition to the canonical TRM marker *CD69*, the CD8^+^ TRM cluster exhibited upregulated expression of classical TRM-associated genes, including the adhesion molecules *ITGAE* (CD103) and *ITGA1* (CD49A) (18, 31); the chemokine receptors *CXCR4* and *CXCR6* (31); regulatory molecules of G protein signaling (*RGS1* and *RGS2*) (18); and the inhibitory markers *LAYN*, *CTLA4*, *HAVCR2* (TIM3), *LAG3*, and *PDCD1* (PD-1) (18, 31) (Figure 2C). Furthermore, gene set enrichment analysis (GSEA) and the Aucell algorithm were used to validate the tissue-residency signature of the CD8^+^ TRM cluster (18, 31, 32) (*P* < 0.0001; Figure 2D and Supplemental Figure 1D). The CD8^+^ TRM cluster also exhibited upregulated pathways involved in GVHD, proinflammation, and T cell proliferation compared with the other circulating counterparts (Figure 2E). The presence of CD8^+^ TRM cells in the intestinal tissue was further confirmed using mIHC staining of CD8 and CD69, which was consistent with the results of previous reports (4, 33). Among patients who received sex-mismatched allo-HSCT and achieved complete donor chimerism status in the peripheral blood, we validated that the majority of intestinal CD8^+^ TRM cells were of donor origin by performing combined mIHC, fluorescence in situ hybridization of X and Y chromosomes (FISH-XY), and souporcell analysis at the scRNA-seq level (Figure 2, F and G, and Supplemental Figure 1E). Therefore, donor-derived CD8^+^ TRM cells are enriched in the intestines of patients with aGVHD.

### GZMK-expressing CD8^+^ TRM subset is associated with intestinal aGVHD.

**Figure 3:**
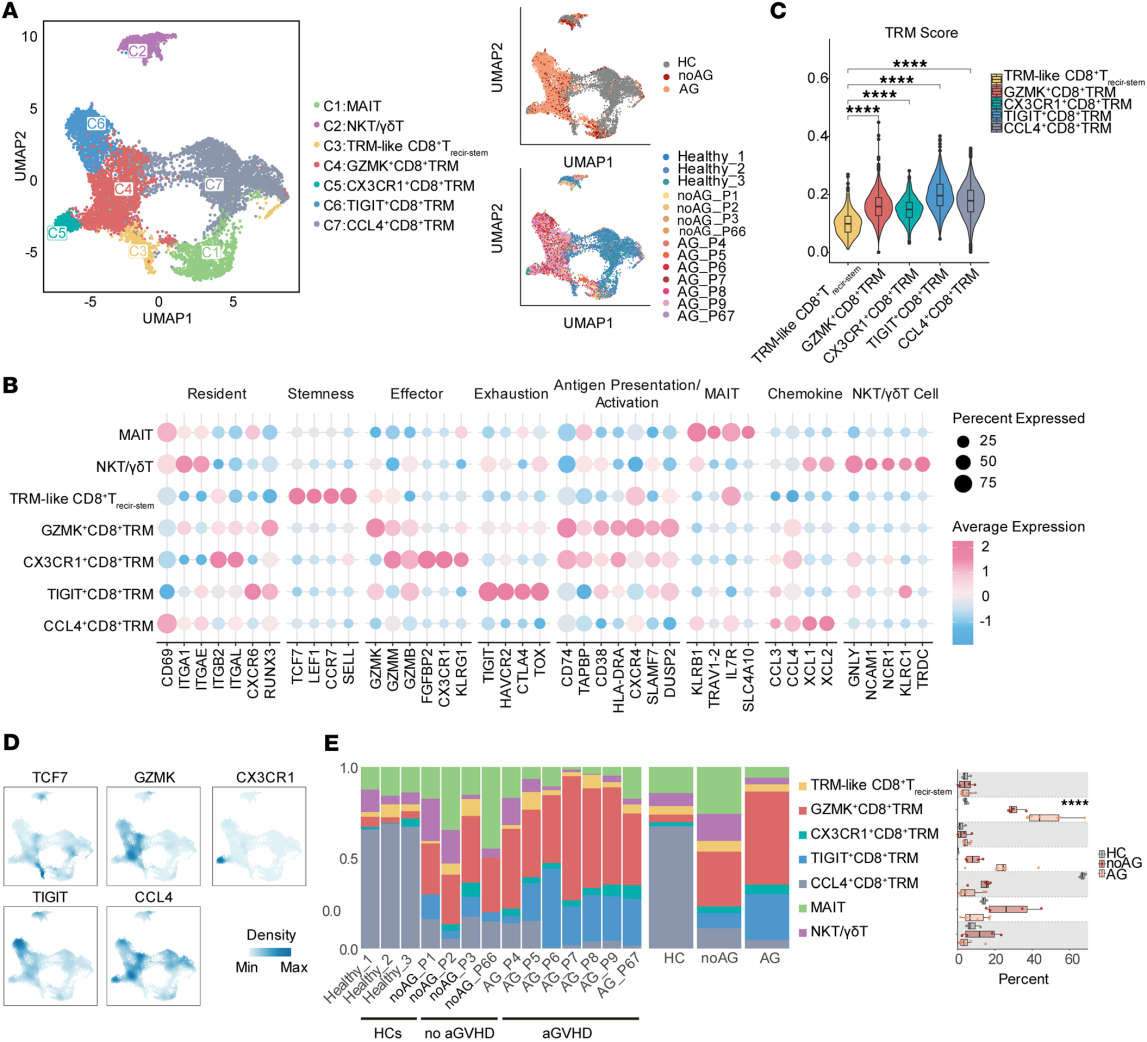
Single-cell analysis identifies distinct CD8^+^ TRM subsets in the human intestine. (**A**) Left: UMAP plot of 9,990 CD8^+^ TRM cells from 3 HCs, 4 patients without aGVHD, and 7 with intestinal aGVHD, which shows 7 CD8^+^ cell subsets according to gene expression. Right: Cells are colored according to 3 groups and 14 samples. (**B**) Bubble plot showing the relative expression of representative genes associated with distinct features of CD8^+^ cell subsets. The color of each dot represents the average normalized expression, ranging from high (red) to low (blue), and the size represents the percentage of positive cells for each gene. (**C**) Violin plot of TRM scores that were calculated for 5 CD8^+^ TRM cell subsets. ****P < 0.0001 according to the Wilcoxon test. (**D**) Feature plots showing the density of representative genes in 5 CD8^+^ cell subsets. (**E**) Left: Stacked bar plot showing the proportions of CD8^+^ TRM cell subsets in each sample and merged groups. Right: CD8^+^ TRM cell subset abundance differences among HCs, no aGVHD, and aGVHD groups. ****P < 0.0001 according to the Wilcoxon test.

**Figure 4:**
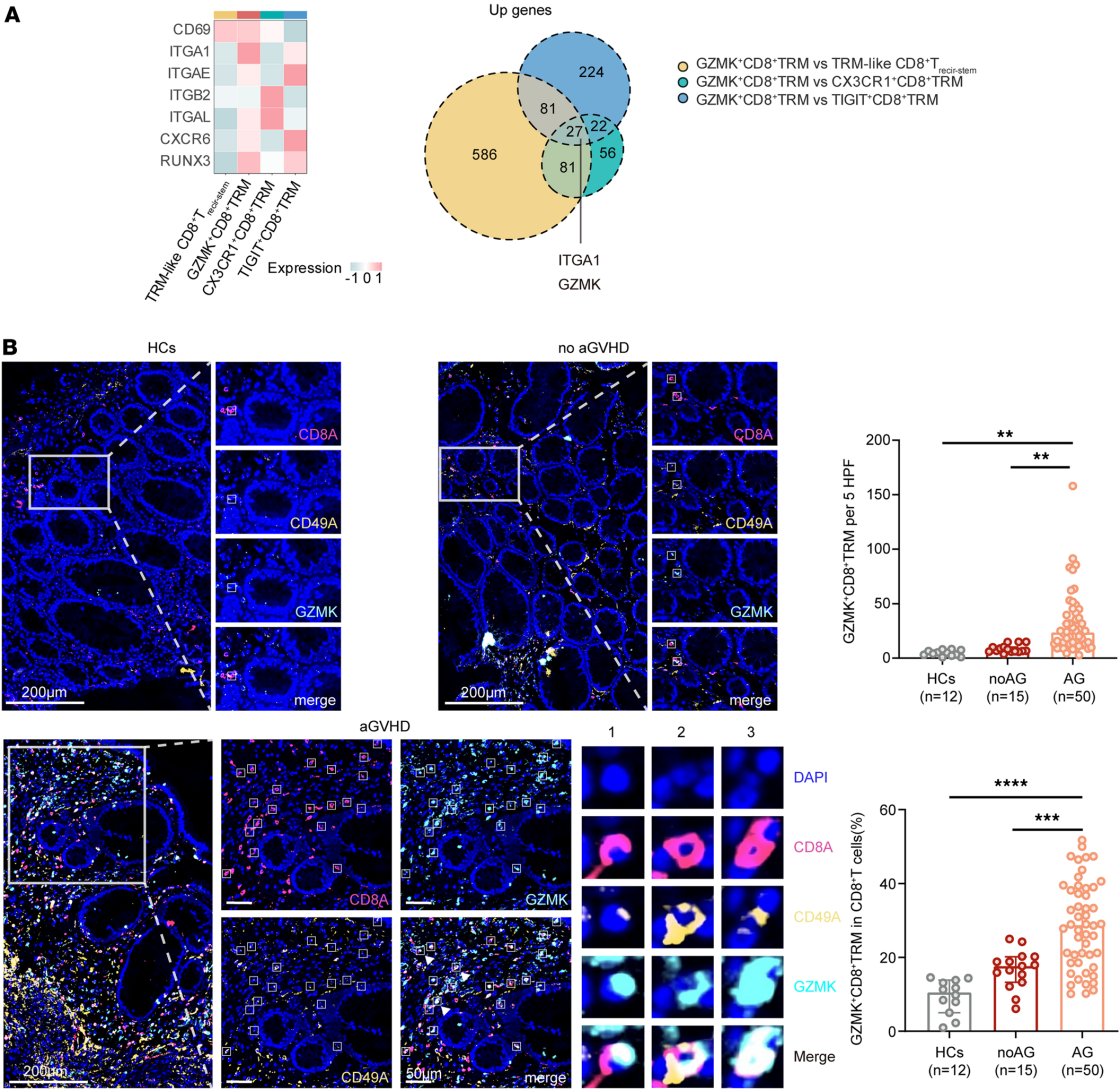
GZMK-expressing CD8^+^ TRM subset is associated with intestinal aGVHD. (**A**) Left: Heatmap showing representative gene expression associated with residency features in 4 CD8^+^ TRM cell subsets. Right: Venn diagram of DEGs that were upregulated in the GZMK^+^CD8^+^ TRM subset compared with the TRM-like CD8^+^ T_recir-stem_, CX3CR1^+^CD8^+^ TRM, and TIGIT^+^CD8^+^ TRM subsets. (**B**) Left: Representative images from intestinal samples subjected to multiplex immunohistochemical (mIHC) staining for CD8, CD49A, and GZMK, as well as DAPI. The white box indicates CD8^+^CD49A^+^GZMK^+^ TRM subset cells. Right panels highlight co-detection in individual cells. Scale bars: 200 μm and 50 μm (zoomed-in images). Right: Distribution of the GZMK^+^CD8^+^ TRM subset in HCs (*n* = 12), no aGVHD (*n* = 15), and patients with intestinal aGVHD (*n* = 50). **P < 0.01; ***P < 0.001; ****P < 0.0001 according to the Wilcoxon test.

We performed further analyses of CD8^+^ T cells from 11 intestinal biopsy samples after allo-HSCT (7 with intestinal aGVHD and 4 without intestinal aGVHD) to characterize the intrinsic structure and potential functional subsets of CD8^+^ T cells. As an untransplanted HC cohort, we integrated a published dataset of 3 healthy individuals from the human gut CD8^+^ TRM cell atlas (34). We identified 7 distinct clusters according to the gene expression profile and paired VDJ profile (Figure 3A and Supplemental Figure 2A). The first cluster, which was the mucosal-associated invariant T (MAIT) cell cluster, expressed high levels of the MAIT marker genes *KLRB1* (CD161), *TRAV1-2*, *IL7R* (CD127), and *SLC4A10* (35, 36). The second cluster, which was an NKT/γδ T cell cluster, expressed high levels of *GNLY*, *NCAM1*, *NCR1*, *KLRC1*, and *TRDC* with a TRM phenotype (Figure 3B) (36, 37). Based on previous published markers, the TRM cells were stringently transcriptomically defined by (a) intrinsically high expression of tissue retention molecules (*CD69*, *ITGA1* [CD49A], *ITGAE* [CD103], *ITGB2* [CD18], *ITGAL* [LFA-1]); (b) the absence of receptors necessary for lymph node homing (*SELL* [CD62L], *CCR7*); (c) upregulation of specific chemokines and chemokine receptors (*CXCR6*); and (d) genes encoding TRM markers (*RUNX3*) (11, 31, 38, 39). After the strict redefinition and further TRM scoring (Figure 3C), the third cluster, which was TRM-like recirculating-stem CD8^+^ T cell (TRM-like CD8^+^ T_recir-stem_) cluster, specifically expressed “stemness” marker genes such as *TCF7*, *LEF1*, *CCR7*, and *SELL* (40, 41). The fourth cluster, which was the GZMK-expressing CD8^+^ TRM (GZMK^+^CD8^+^ TRM), was characterized by high expression of *GZMK*, *ITGA1*, *GZMM*, and *CXCR4* (42-45). The fifth cluster, which was the CX3CR1-expressing CD8^+^ TRM (CX3CR1^+^CD8^+^ TRM) cluster, expressed high levels of the effector marker genes *CX3CR1*, *GZMB*, *FGFBP2*, and *KLRG1*. The sixth cluster, which was the TIGIT-expressing CD8^+^ TRM (TIGIT^+^CD8^+^ TRM) cluster, expressed high levels of the exhausted marker genes *TIGIT*, *HAVCR2* (TIM3), *CTLA4*, and *TOX* (42, 44). The seventh cluster, which was the CCL4-expressing TRM (CCL4^+^CD8^+^ TRM) cluster, expressed high levels of *CCL3*, *CCL4*, *XCL1*, and *XCL2*. (Figure 3, B and D, Supplemental Figure 2, B and C, and Supplemental Table 3). For subsequent CD8^+^ TRM subset analysis, we did not focus on the CCL4^+^CD8^+^ TRM subset, because the CCL4^+^CD8^+^ TRM subset was mainly present in untransplanted HCs, but little in patients with or without intestinal aGVHD (Figure 2E). Compared with the other subsets, the GZMK^+^CD8^+^ TRM subset was the highest proportion subset and specifically expressed higher levels of *ITGA1* (CD49A) and *GZMK* (Figure 3E and Supplemental Figure 2D). Based on its marker genes, we identified the GZMK^+^CD8^+^ TRM subset as CD8^+^GZMK^+^CD49A^+^, and the GZMK^+^CD8^+^ TRM subset demonstrated expression of tissue retention molecule CD69 but little CD103, TCF1, CCR7, and CD62L (Figure 4A, Supplemental Figure 2, E and F, and Supplemental Table 4). We further performed mIHC on intestinal biopsy samples from 65 patients after allo-HSCT (50 patients with intestinal aGVHD and 15 without intestinal aGVHD) and 12 HCs. Compared with those in the HCs and patients without intestinal aGVHD, the mIHC findings in intestinal aGVHD patients revealed greater numbers and ratios of GZMK^+^CD8^+^ TRM subset infiltration (*P* < 0.01 and *P* < 0.001; Figure 4B). The numbers and ratios of GZMK^+^CD8^+^ TRM subset infiltration were also significantly increased in steroid refractory groups (Supplemental Figure 2G). Multivariable binary logistic regression analysis further showed that the number of GZMK^+^CD8^+^ TRM cells was associated with the diagnosis of intestinal aGVHD (Table 2). Our analysis revealed that intestinal aGVHD is associated with infiltration of the intestine with GZMK^+^CD8^+^ T cells with TRM features.

### GZMK^+^CD8^+^ TRM subset exhibits enhanced clonal expansion and IFN signaling pathway–associated proinﬂammatory properties in intestinal aGVHD.

**Figure 5:**
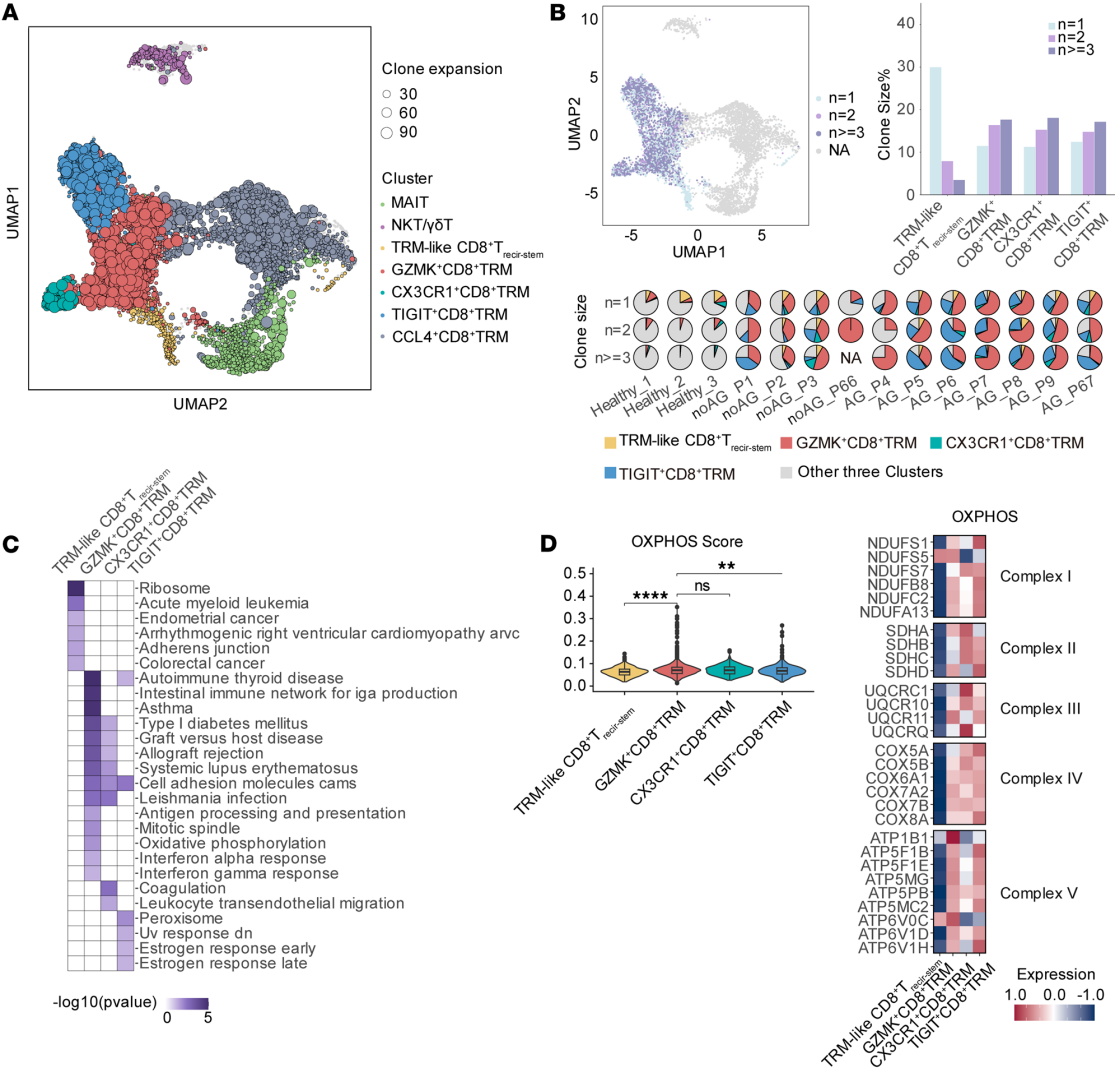
GZMK^+^CD8^+^ TRM subset shows enhanced clonal expansion and upregulated proinflammatory pathway expression in intestinal aGVHD. (**A**) UMAP embedding of the scRNA-seq data of T cells that were mapped to TCR clone information, color-coded according to CD8^+^ TRM cell subsets. Clonal expansion, which was defined by a shared CDR3 nucleotide sequence and V and J gene usage, is depicted according to marker size. (**B**) Top: UMAP and bar plot showing percentage of TRM-like CD8^+^ T_recir-stem_, GZMK^+^CD8^+^ TRM, CX3CR1^+^CD8^+^ TRM, and TIGIT^+^CD8^+^ TRM subsets stratified according to clone size. Bottom: Pie charts showing the CD8^+^ TRM cell subset composition of the clones of each sample, stratified according to clone size in individual samples. (**C**) GSEA of enriched pathways (KEGG and HALLMARK) for 4 CD8^+^ TRM cell subsets (*P* value < 0.05). (**D**) Violin plot of enrichment scores (left) and heatmap of representative genes for the oxidative phosphorylation (OXPHOS) signature in 4 CD8^+^ TRM cell subsets (right). **P < 0.01; ****P < 0.0001 according to the Wilcoxon test.

**Figure 6:**
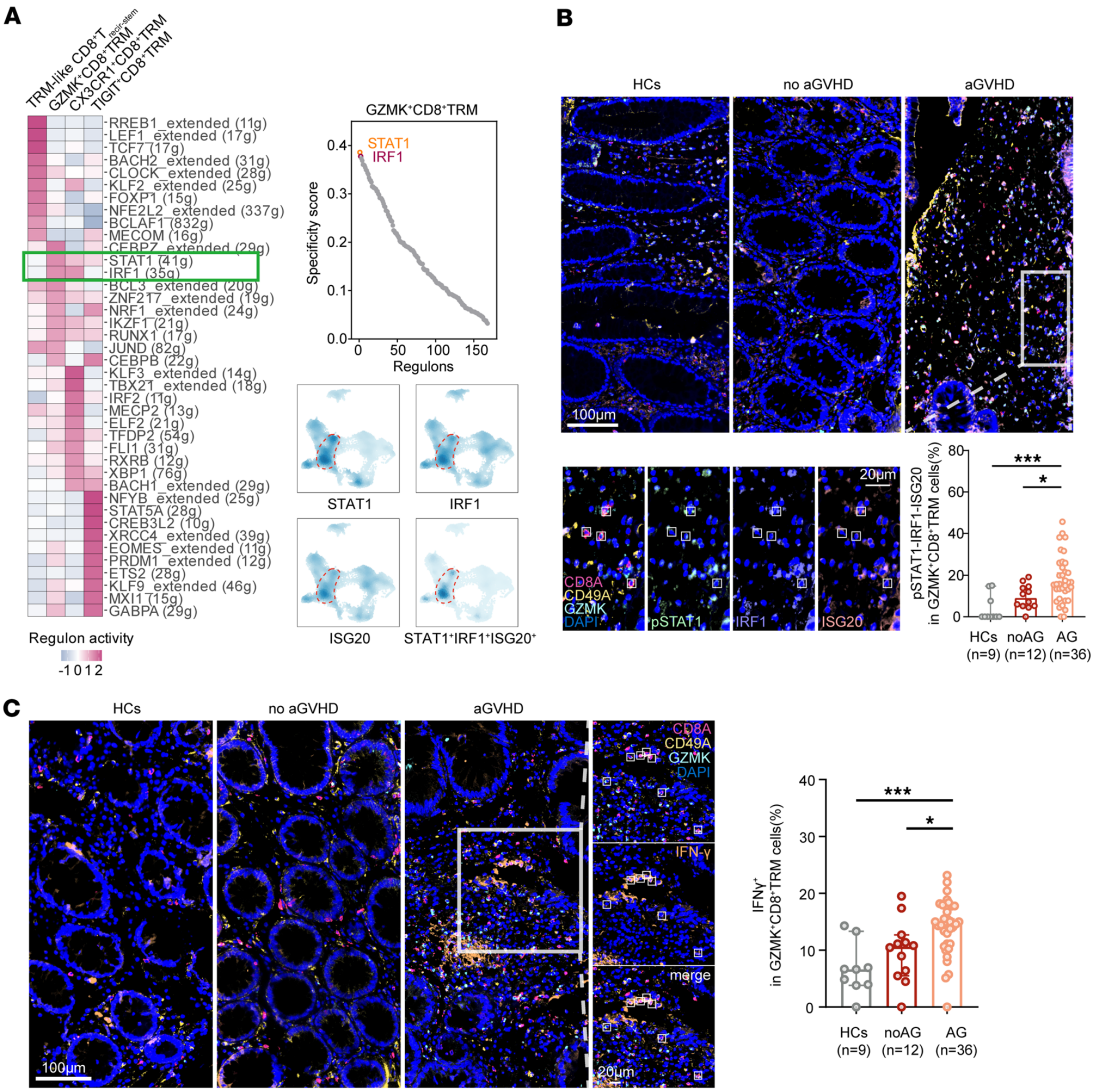
GZMK^+^CD8^+^ TRM cells upregulated STAT1/IRF1-related inflammatory signaling in intestinal aGVHD. (**A**) Left: Heatmap showing the top 10 scaled transcription factor activities in 4 CD8^+^ TRM cell subsets, as calculated by SCENIC analysis. Right: Specificity of regulons in GZMK^+^CD8^+^ TRM cells based on the regulon specificity score (RSS). The colors indicate the most specific IRF1 and STAT1 regulons. (**B**) Left: Representative mIHC staining images of CD8, CD49A, GZMK, p-STAT1, IRF1, and ISG20, as well as DAPI. Right: Proportions of p-STAT1, IRF1, and ISG20 coexpressed in the GZMK^+^CD8^+^ TRM cell subset (HCs, *n* = 9; no aGVHD, *n* = 12; aGVHD, *n* = 36). (**C**) Left: Representative mIHC staining images of CD8, CD49A, GZMK, IFN-γ, as well as DAPI. Right: Proportions of IFN-γ in the CD8^+^GZMK^+^ TRM cell subset (HCs, *n* = 9; no aGVHD, *n* = 12; aGVHD, *n* = 36). Scale bars: 100 μm and 20 μm (zoomed-in images). *P < 0.05; ***P < 0.001 according to the Wilcoxon test.

We mapped the TCR clone information to UMAP to visualize the clone size and distribution across CD8^+^ TRM subsets (Figure 5A). The CD8^+^ TRM subsets exhibited a diverse TCR repertoire, ranging from moderately clonally expanded (*n* = 2) to highly clonally expanded (*n* ≥ 3). Expanded clones showed a nonuniform distribution of CD8^+^ TRM subsets (Supplemental Figure 3A). Furthermore, the GZMK^+^CD8^+^ TRM subset exhibited enhanced clonal expansion (Figure 5B and Supplemental Figure 3B). We further explored the function of the different CD8^+^ TRM subsets. GSEA of enriched pathways revealed that the upregulated DEGs in the GZMK^+^CD8^+^ TRM subset were associated with GVHD, the IFN-γ response, and antigen processing/presentation signaling pathways compared with other CD8^+^ TRM subsets (Figure 5C and Supplemental Figure 3C). Moreover, the GZMK^+^CD8^+^ TRM subset exhibited the highest oxidative phosphorylation (OXPHOS) score and was highly enriched in ATP-related pathways (Figure 5D and Supplemental Figure 3D). The transcriptional regulatory network that was inferred by SCENIC based on scRNA-seq data confirmed that the transcription factor activities of *STAT1* and *IRF1* were greater in the GZMK^+^CD8^+^ TRM subset. Based on the regulon specificity score (RSS), *STAT1* and *IRF1* were identified as being the most specific regulons in the GZMK^+^CD8^+^ TRM subset (Figure 6A). We compared the atlas-wide similarity of activity scores for every regulon pair using the Connection Specificity Index (CSI). The results demonstrated that *STAT1* and *IRF1* are localized in one common major module, thus suggesting that they specifically coregulate the GZMK^+^CD8^+^ TRM subset (Supplemental Figure 3E). In addition, the transcriptomic expression levels of *STAT1*, *IRF1*, and downstream ISG-related genes (such as *ISG20*, which are enriched in the IFN signaling pathway) were increased (Figure 6B and Supplemental Figure 2B). Compared with HCs and patients without intestinal aGVHD, p-STAT1, IRF1, and ISG20 were significantly upregulated in the GZMK^+^CD8^+^ TRM subset of intestinal aGVHD patients according to mIHC (*P* < 0.05; Figure 3F). Additionally, GZMK^+^CD8^+^ TRM cells from aGVHD patients secreted more IFN-γ (*P* < 0.05; Figure 6C). Our findings indicate that GZMK^+^CD8^+^ TRM cells are associated with intestinal aGVHD and display enhanced clonal expansion and IFN signaling–associated proinflammatory pathway expression.

### GZMK^+^CD8^+^ TRM subset displayed lineage bifurcation differentiation properties and differentiated more into an effector-like CD8^+^ TRM subset in intestinal aGVHD.

**Figure 7:**
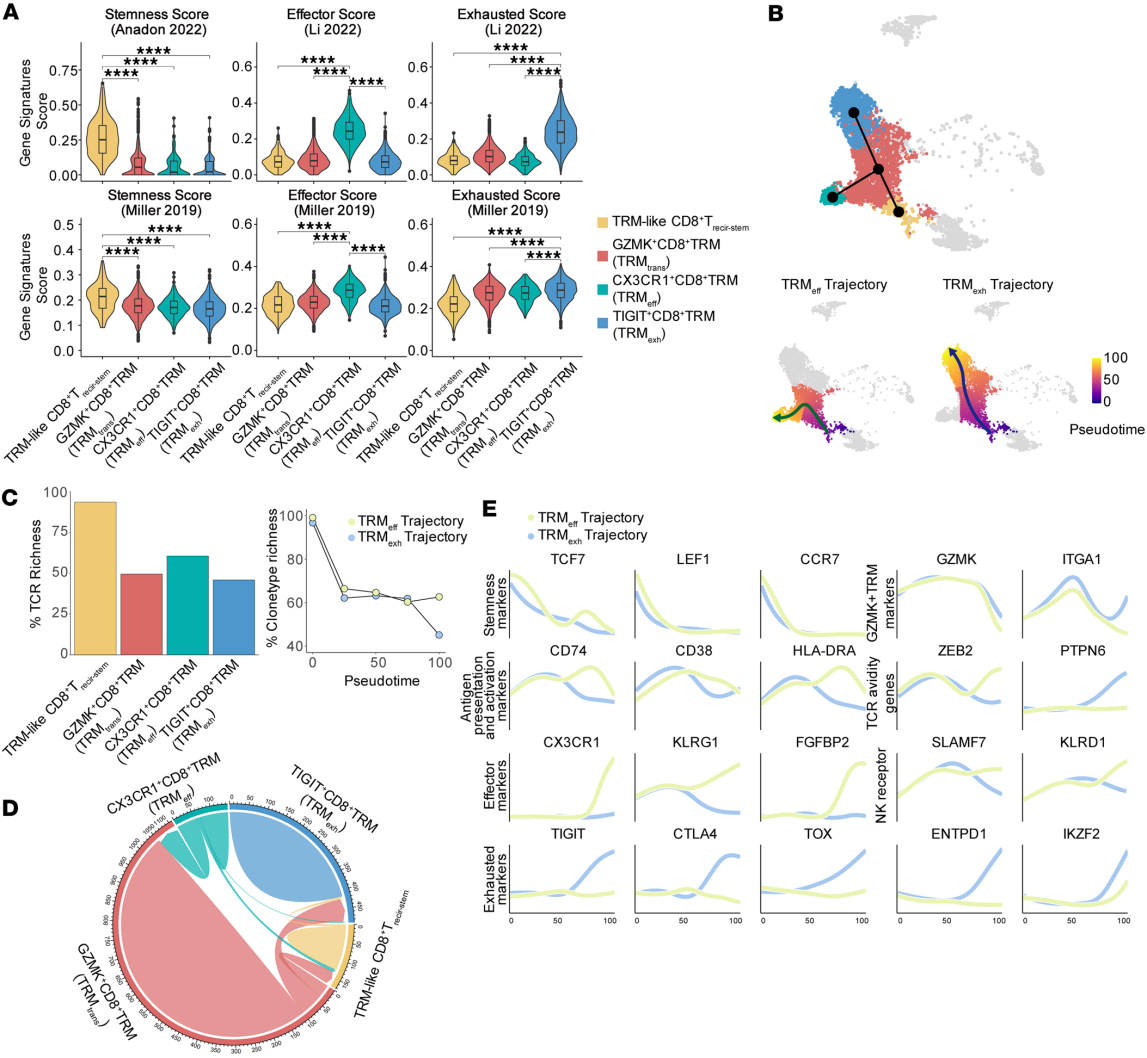
GZMK^+^CD8^+^ TRM cells display lineage bifurcation differentiation properties. (**A**) Violin plot of enrichment scores for the stemness, effector, and exhausted signatures in 4 CD8^+^ TRM cell subsets. ****P < 0.0001 according to the Wilcoxon test. (**B**) Pseudotime trajectories for CD8^+^ TRM cell subsets based on Slingshot, which show 2 trajectories (effector-like TRM [TRM_eff_] and exhausted-like TRM [TRM_exh_]), color-coded for CD8^+^ TRM cell subsets (left) and pseudotime (right). (**C**) Left: TCR richness for CD8^+^ TRM cell subsets. Right: Clonotype richness along the CD8^+^ TRM_eff_ and CD8^+^ TRM_exh_ trajectories. (**D**) Chord diagram of shared TCR clones for the 4 CD8^+^ TRM cell subsets. (**E**) Plot of markers and functional genes along the CD8^+^ TRM cell trajectories.

**Figure 8:**
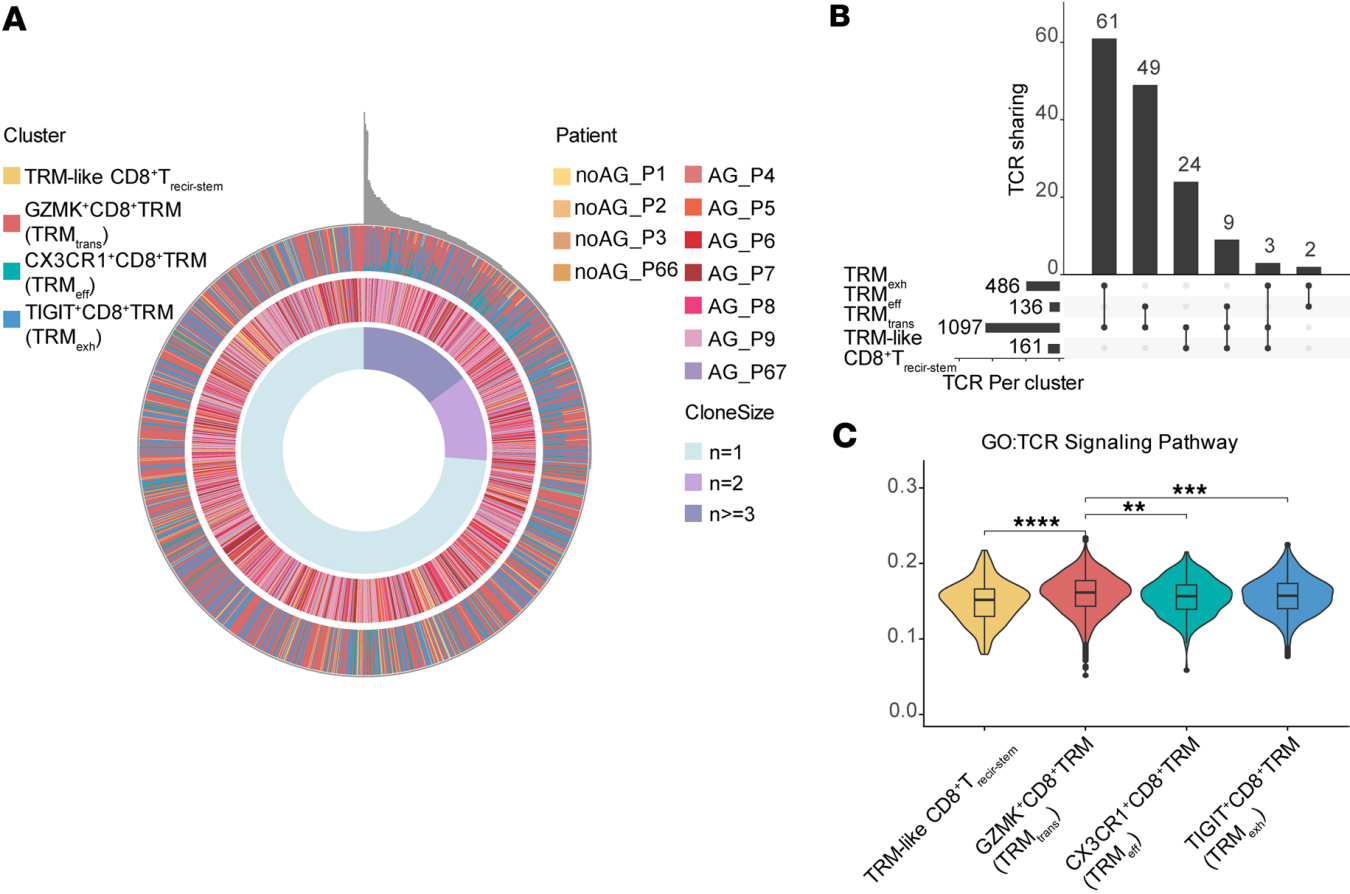
GZMK^+^CD8^+^ TRM cells show clonal sharing and enhanced TCR signaling. (**A**) Circos plot of combined TCR sequencing and transcriptomic data. The outer ring (gray bar graphs) indicates the TCR frequency (1 bar per clonotype). The second ring is colored by the CD8^+^ TRM cell subset. The third ring is colored by the individual intestinal samples. The inner ring is colored by the clone size. (**B**) UpSet plot showing the degree of shared TCR clones among CD8^+^ TRM cell subsets. (**C**) Violin plot of enrichment scores for the TCR signaling pathway signature (GO) in 4 CD8^+^ TRM cell subsets. **P < 0.01; ***P < 0.001; ****P < 0.0001 according to the Wilcoxon test.

**Figure 9:**
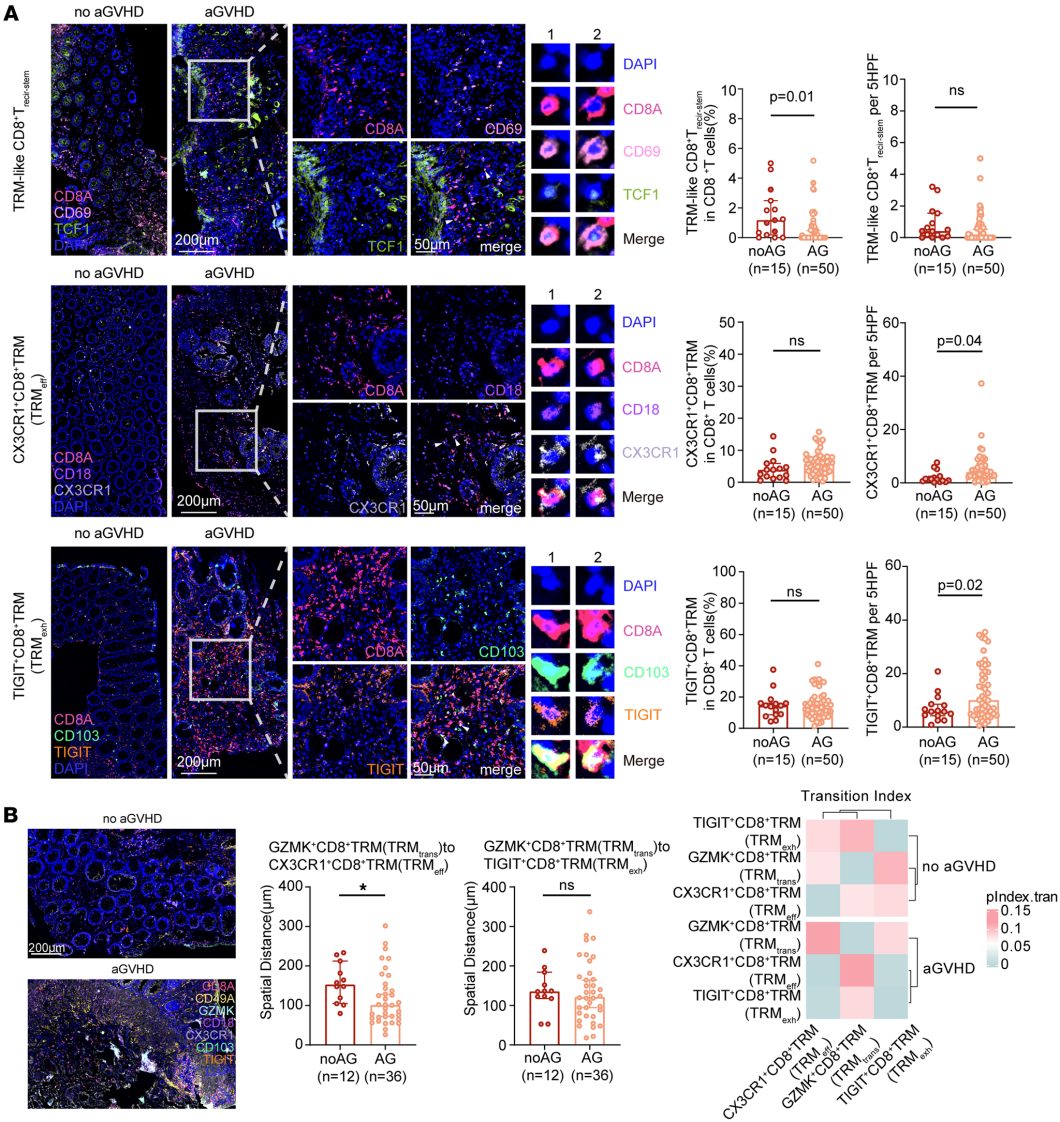
Spatial analysis shows preferential differentiation of GZMK^+^CD8^+^ TRM cells toward effector-like TRM cells in intestinal aGVHD. (**A**) Left: Representative multiplex immunohistochemical (mIHC) staining images for CD8, CD69, TCF1, CD18, CX3CR1, CD103, and TIGIT, as well as DAPI of the intestinal samples. The white boxes indicate TRM-like CD8^+^ T_recir-stem_, TRM_eff_, and TRM_exh_ subsets. Scale bars: 200 μm and 50 μm (zoomed-in images). Right: Percentages and numbers of the TRM-like CD8^+^ T_recir-stem_, TRM_eff_, and TRM_exh_ subsets (no aGVHD, *n* = 15; aGVHD, *n* = 50). (**B**) Left: Representative mIHC staining images for CD8, CD49A, GZMK, CD18, CX3CR1, CD103, and TIGIT, as well as DAPI of intestinal samples. Spatial distances between different CD8^+^ TRM subsets. Scale bar: 200 μm. Right: Heatmap of transition index in the different CD8^+^ TRM cell subsets (no aGVHD, *n* = 12; aGVHD, *n* = 36). *P < 0.05 according to Student’s *t* test.

After scoring for stemness, effector, and exhausted signatures (32, 43, 46), the TRM-like CD8^+^ T_recir-stem_, CX3CR1^+^CD8^+^ TRM, and TIGIT^+^CD8^+^ TRM subsets displayed significant expression of stemness, effector, and exhausted gene signatures, respectively. The GZMK^+^CD8^+^ TRM subset exhibited moderate expression of both effector and exhausted gene signatures and a low expression of the stemness gene signature, thus suggesting that this subset has features of transition (*P* < 0.0001; Figure 7A). To infer potential differentiation relationships, we observed 2 distinct CD8^+^ TRM subset pseudotime trajectories using Slingshot (47), PAGA (48), and Monocle3 (49) (Figure 7B and Supplemental Figure 4, A and B). The TRM-like CD8^+^ T_recir-stem_ cluster had the highest TCR richness, which we considered to be the root of the trajectories (Figure 7C). The TRM-like CD8^+^ T_recir-stem_ cluster differentiated into the GZMK^+^CD8^+^ TRM cluster. The GZMK^+^CD8^+^ TRM cluster, referred to as transition-like TRM (TRM_trans_), branched into 2 different trajectories to form the CX3CR1^+^CD8^+^ TRM (effector-like TRM, TRM_eff_) and TIGIT^+^CD8^+^ TRM (exhausted-like TRM, TRM_exh_) clusters. The decrease in TCR richness along the trajectories further confirmed the differentiation trajectories (Figure 7C). Contiguous CD8^+^ TRM subsets within the trajectories shared TCR clonotypes, and the TRM_trans_ cluster was able to differentiate into both TRM_eff_ and TRM_exh_ clusters (Figure 7D and Supplemental Figure 4C), which further supported the plausibility of these inferred trajectories. The DEGs and gene signature enrichment scores along 2 pseudotime trajectories demonstrated that the functions of different CD8^+^ TRM cell subsets are diverse. Along 2 pseudotime trajectories, stemness-related genes (*TCF7*, *LEF1*, and *CCR7*) were downregulated, whereas effector-related (*KLRG1* and *FGFBP2*) and exhausted-related genes (*CTLA4* and *TOX*) were upregulated, in the TRM_eff_ and TRM_exh_ trajectories, respectively (32, 43). As previously reported, *ZEB2* and *PTPN6* (SHP-1) were upregulated in the TRM_eff_ and TRM_exh_ trajectories, respectively, further indicating that CD8^+^ TRM differentiation is related to TCR affinity and signal strength (42). In addition, the NK cell receptor genes *SLAMF7* and *KLRD1* were upregulated in the TRM_eff_ trajectories, and *ENTPD1* and *IKZF2* were upregulated in the TRM_exh_ trajectories. In the TRM_trans_ cluster, the antigen presentation–related marker *CD74*, the T cell activation–related marker *CD38*, and *HLA-DRA* were upregulated at early time points and remained the most highly expressed genes in the TRM_eff_ trajectory. The GZMK expression levels were maximal in the TRM_trans_ cluster and progressively decreased during the TRM_eff_ and TRM_exh_ trajectories (Figure 7E and Supplemental Figure 4D). Visualization of the combination of transcriptional cell states with their clonal frequency for CD8^+^ TRM subsets revealed that the TRM_trans_ cluster exhibited a high degree of cell state sharing (Figure 8A). Furthermore, the UpSet plot revealed that 80% of the expanded clones in the TRM_trans_ cluster lacked TRM-like CD8^+^ T_recir-stem_ counterparts (Figure 8B). To understand whether the observed clonal expansion in the TRM_trans_ subset could be due to the TCR signal, we demonstrated that TCR signaling pathway scores were highest in the TRM_trans_ subset (Figure 8C). Based on the DEGs among the CD8^+^ TRM subsets, we further identified the TRM-like CD8^+^ T_recir-stem_ subset as CD8^+^CD69^+^TCF1(TCF7)^+^, the TRM_eff_ subset as CD8^+^CD18(ITGB2)^+^CX3CR1^+^, and the TRM_exh_ subset as CD8^+^CD103(ITGAE)^+^TIGIT^+^. Our mIHC staining results showed that there was a lower tendency for infiltration of the TRM-like CD8^+^ T_recir-stem_ subset in intestinal aGVHD patients (Figure 9A). Although the TRM_eff_ and TRM_exh_ subset infiltration increased, it was not significantly increased compared with TRM_trans_ subset infiltration (*P* = 0.04 and *P* = 0.02; Figure 9A). We subsequently compared the spatial distances among the CD8^+^ TRM subsets. We found that the TRM_trans_ subset in aGVHD patients exhibited a greater proximity to the TRM_eff_ compared with the TRM_exh_ subset (*P* < 0.05; Figure 9B). Transition index analysis also further suggested that the TRM_trans_ subset differentiated more into a TRM_eff_ subset in intestinal aGVHD (Figure 9B). Our findings indicated that the GZMK^+^CD8^+^ TRM subset displayed lineage bifurcation differentiation properties and differentiated more into a CD8^+^ TRM_eff_ subset in intestinal aGVHD.

### High infiltration of the GZMK^+^CD8^+^ TRM subset correlates with the severity of intestinal aGVHD and predicts the outcome of patients with intestinal aGVHD.

**Figure 10:**
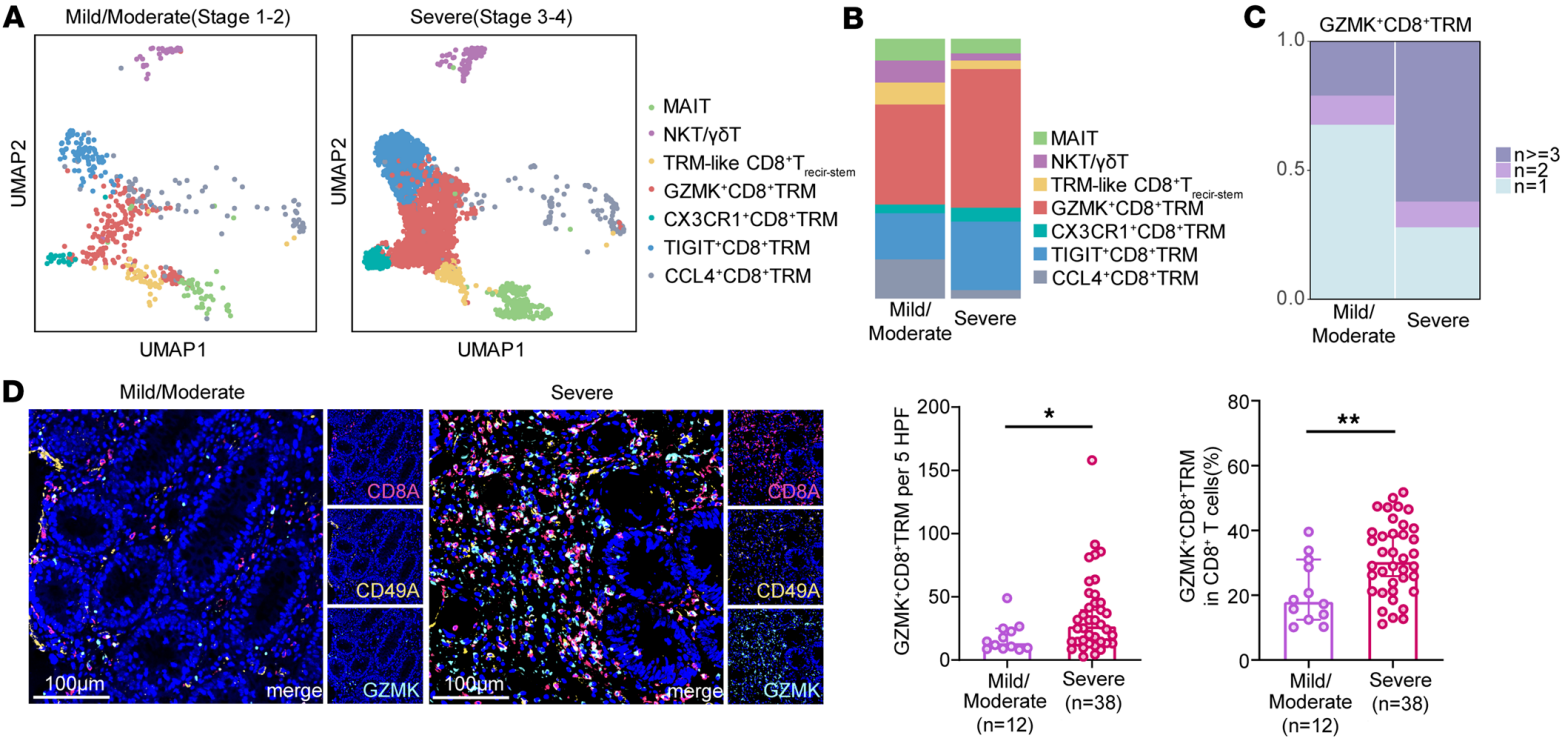
GZMK^+^CD8^+^ TRM cell infiltration correlates with intestinal aGVHD severity. (**A**) UMAP plot of intestinal CD8^+^ TRM cells from mild/moderate (stages 1–2) (582 cells) and severe (stages 3–4) (3,914 cells) intestinal aGVHD patients. (**B**) Stacked bar plot of CD8^+^ TRM subset cells in mild/moderate and severe intestinal aGVHD patients. (**C**) Quantification of clone size for CD8^+^ TRM subset cells in mild/moderate and severe intestinal aGVHD patients. (**D**) Representative images (left) and quantification (right) of GZMK^+^CD8^+^ TRM subset cells according to GVHD severity. Percentages and numbers of the GZMK^+^CD8^+^ TRM subset cells in mild/moderate (*n* = 12) and severe (*n* = 38) intestinal aGVHD patients. Scale bars: 100 μm. *P < 0.05; **P < 0.01 according to Student’s *t* test.

**Figure 11:**
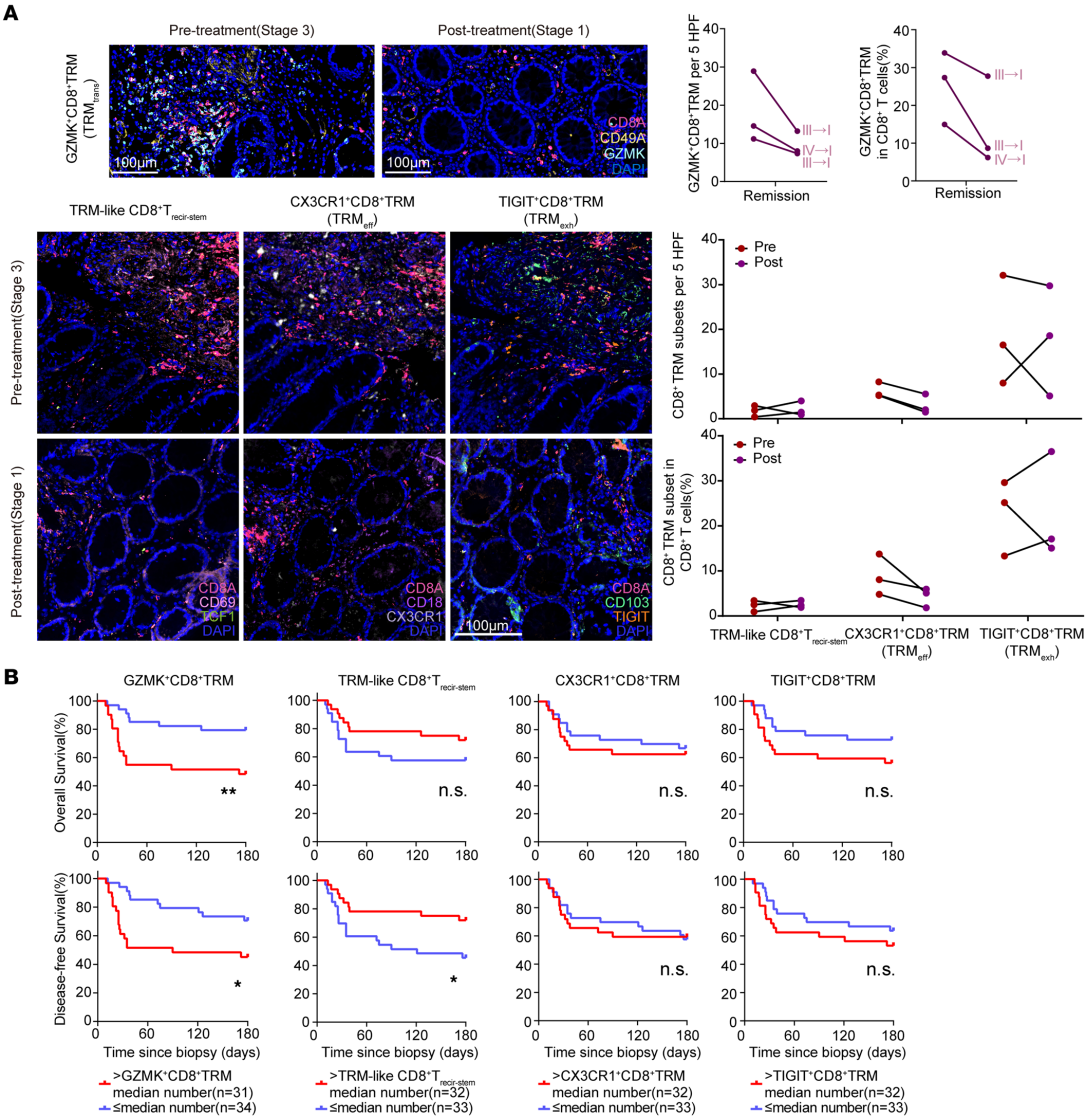
GZMK^+^CD8^+^ TRM cell infiltration decreases after remission and predicts poor survival. (**A**) Left: Representative images of GZMK^+^CD8^+^ TRM cells from one patient after remission (remission from stage 3 to stage 1). Scale bars: 100 μm. Right: The changes in the numbers and percentages of GZMK^+^CD8^+^ TRM, TRM-like CD8^+^ T_recir-stem_, TRM_eff_, and TRM_exh_ subset cells during the remission process (*n* = 3). (**B**) Overall survival (OS) and disease-free survival (DFS) are associated with the absolute number of infiltrating of GZMK^+^CD8^+^ TRM, TRM-like CD8^+^ T_recir-stem_, TRM_eff_, and TRM_exh_ subset cells at the time of biopsy in intestinal aGVHD patients, as assessed via mIHC. *P < 0.05; **P < 0.01 according to the 2-sided log-rank (Mantel-Cox) test.

We further explored whether high infiltration of the GZMK^+^CD8^+^ TRM subset was associated with intestinal aGVHD severity. We grouped the intestinal aGVHD samples into mild/moderate (stages 1–2) and severe (stages 3–4) according to modified Glucksberg criteria (50) and compared the frequency distributions of TRM subsets between the 2 groups (Figure 10A). Compared with the mild/moderate aGVHD group, the frequency of the GZMK^+^CD8^+^ TRM subset was greater in the severe aGVHD group (Figure 10B). Moreover, the GZMK^+^CD8^+^ TRM subset in the severe aGVHD group was highly clonally expanded (Figure 10C). Compared with 12 mild/moderate aGVHD patients, a significant increase in the GZMK^+^CD8^+^ TRM subset of 38 severe aGVHD patients was detected via mIHC (*P* < 0.05 and *P* < 0.01; Figure 10D). Moreover, GZMK^+^CD8^+^ TRM subset infiltration decreased as aGVHD improved after treatment (Figure 11A). In contrast, the infiltration of the CX3CR1^+^ TRM_eff_ subset showed a decreasing trend, while TRM-like CD8^+^ T_recir-stem_ and TIGIT^+^ TRM_exh_ subsets did not change significantly after treatment (Figure 11A). Survival analysis demonstrated that high GZMK^+^CD8^+^ TRM subset infiltration was associated with worse overall survival (OS) and disease-free survival (DFS) (*P* < 0.01 and *P* < 0.05; Figure 11B). However, CX3CR1^+^ TRM_eff_ and TIGIT^+^ TRM_exh_ infiltration was not associated with survival and prognosis (Figure 11B). Multivariable analysis of risk factors for OS and DFS further showed that absolute cell number of the GZMK^+^CD8^+^ TRM subset was a risk factor for OS, whereas no risk factors were identified for DFS (Table 3). Our data indicate that a higher absolute number of infiltrating GZMK^+^CD8^+^ TRM cells at the time of biopsy was associated with lower subsequent survival.

### Donor-derived GZMK^+^CD8^+^ TRM cell deficiency attenuates intestinal aGVHD severity.

**Figure 12:**
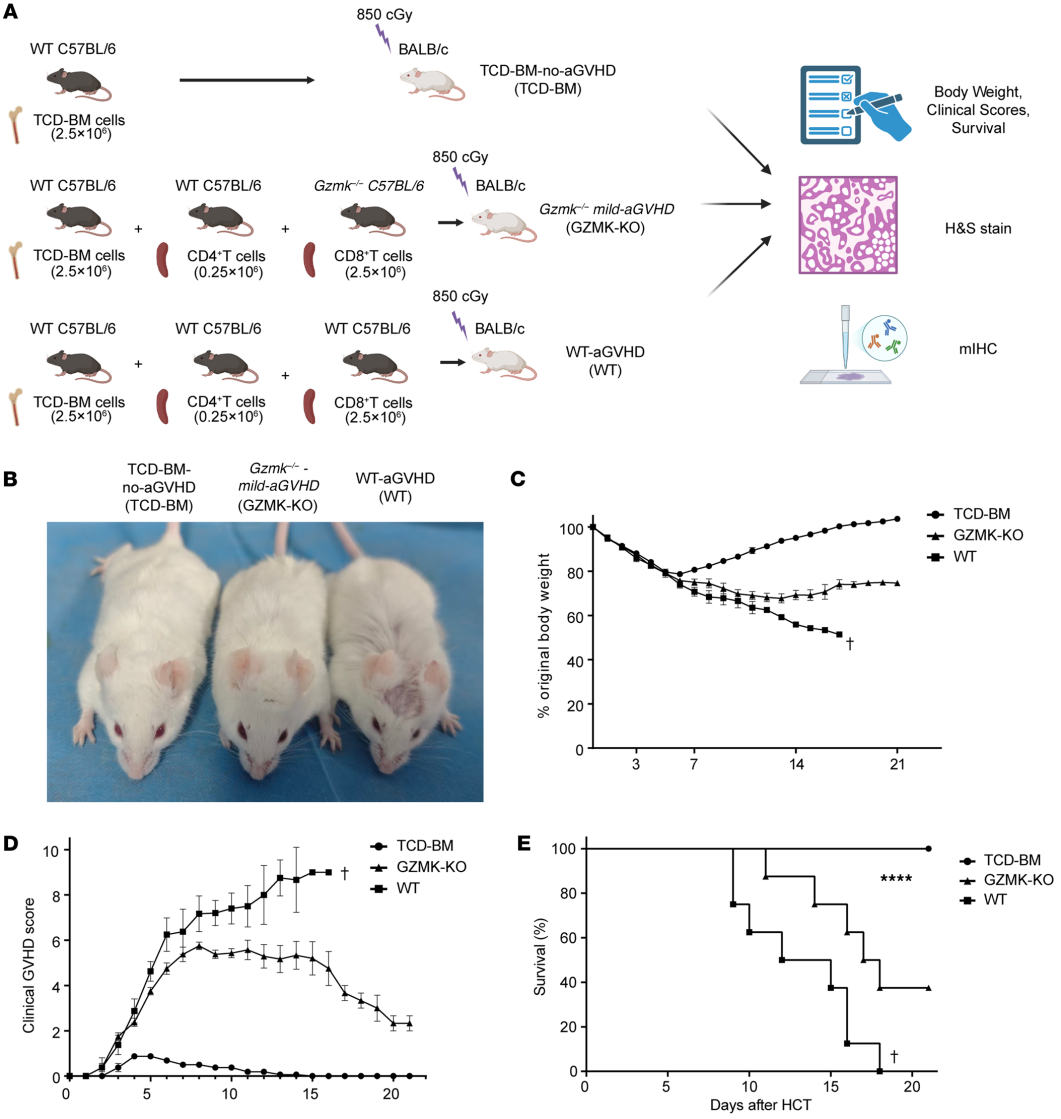
Donor-derived GZMK^+^CD8^+^ TRM cell deficiency attenuates intestinal aGVHD severity. Lethally irradiated BALB/c recipients transplanted with 2.5 × 10^6^ T cell–depleted bone marrow cells (TCD-BM) from WT C57BL/6 donors alone or together with sorted CD4^+^ T cells (0.25 × 10^6^) from WT C57BL/6 donor spleen and sorted CD8^+^ T cells (2.5 × 10^6^) from WT C57BL/6 or *Gzmk^–/–^* C57BL/6 donor spleen. Recipients were monitored for GVHD development, including body weight change, clinical aGVHD scores, and survival. (**A**) The diagram of the experimental design. (**B**) A representative photograph taken at day 14 is shown. (**C**–**E**) Body weight change, clinical aGVHD scores, and survival percentage († indicates death of all recipients in a group); *n* = 8 recipients from 2 replicate experiments. ****P < 0.0001 according to the 2-sided log-rank (Mantel-Cox) test.

**Figure 13:**
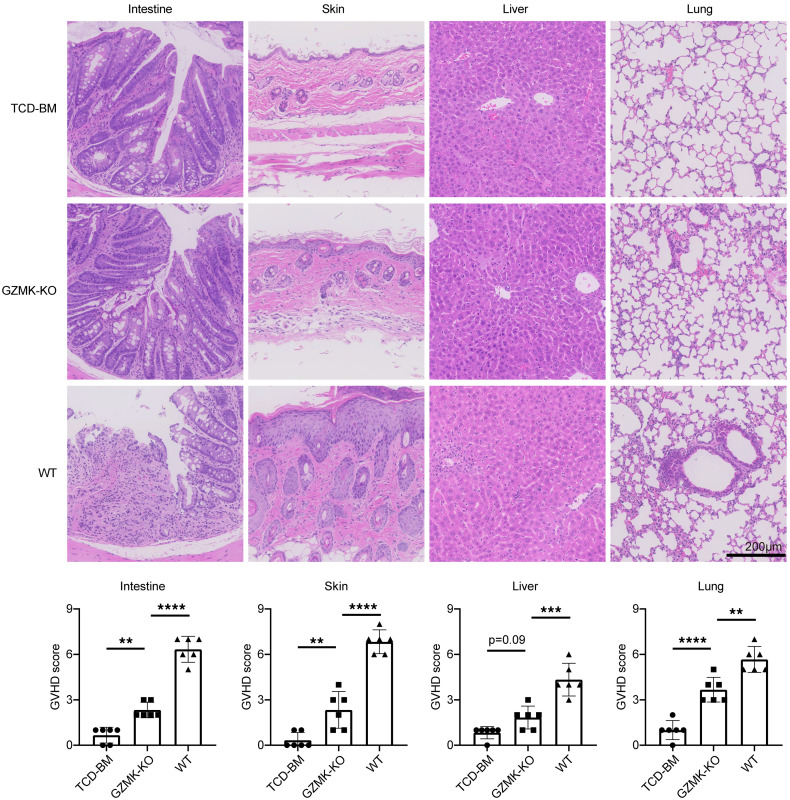
GZMK deficiency alleviates target-organ pathological injury in murine aGVHD. On day 14 after HCT, histopathology of the aGVHD target tissues intestine, skin, liver, and lung was evaluated. A representative photomicrograph (scale bar: 200 μm) and mean ± SEM (*n* = 6) of histopathology scores are shown. **P < 0.01; ***P < 0.001; ****P < 0.0001 according to the Wilcoxon test.

**Figure 14:**
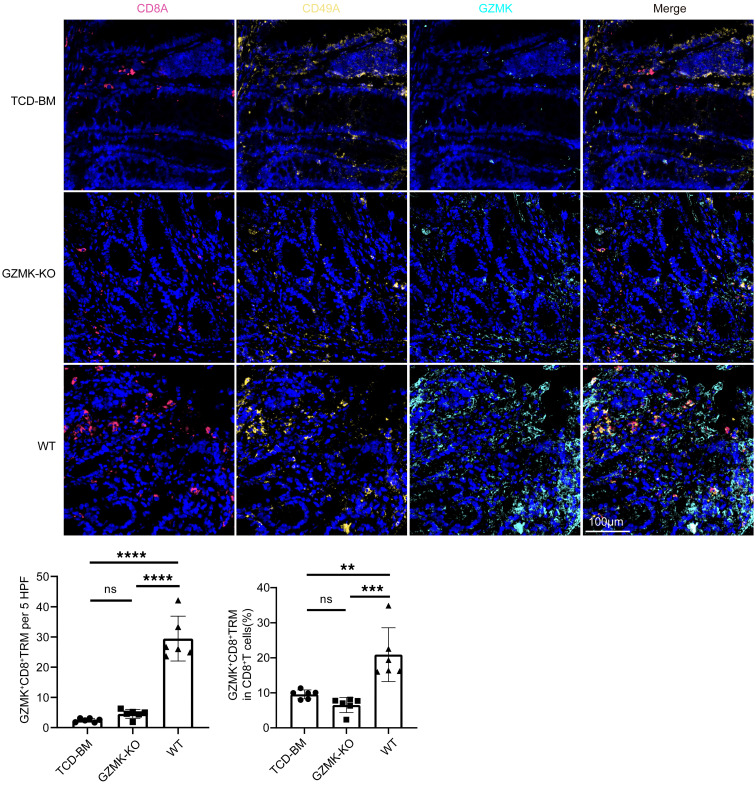
GZMK deficiency reduces intestinal GZMK^+^CD8^+^ TRM cell infiltration in murine aGVHD. (**A**) Representative mIHC staining images for CD8, CD49A, and GZMK, as well as DAPI of intestinal samples. Scale bar: 100 μm. Percentages and numbers of the GZMK^+^CD8^+^ TRM subset in the TCD-BM, GZMK-KO, and WT groups (*n* = 6). **P < 0.01; ***P < 0.001; ****P < 0.0001 according to the Wilcoxon test.

To confirm the role of donor-derived GZMK^+^CD8^+^ TRM cells in intestinal aGVHD, BALB/c recipients were injected with 2.5 × 10^6^ T cell–depleted bone marrow cells (TCD-BM) from wild-type (WT) C57BL/6 donors alone or together with sorted CD4^+^ T cells (0.25 × 10^6^) from WT C57BL/6 donor spleen and sorted CD8^+^ T cells (2.5 × 10^6^) from WT C57BL/6 or *Gzmk^–/–^* C57BL/6 donor spleen (Figure 12A). Recipients given TCD-BM from WT C57BL/6 donors showed no signs of aGVHD and were used as TCD-BM-no-aGVHD recipients. Recipients given sorted CD4^+^ T cells and CD8^+^ T cells from WT C57BL/6 donor spleen developed overt aGVHD with body weight loss, clinical signs, and mortality, and 0% survived for more than 21 days and were described as WT-aGVHD recipients (Figure 12, B–D). In contrast, recipients given sorted CD4^+^ T cells (WT C57BL/6) and sorted CD8^+^ T cells from *Gzmk^–/–^* C57BL/6 donor spleen developed mild aGVHD with fewer clinical signs and less body weight loss, and approximately 40% survived beyond 21 days and were described as *Gzmk^–/–^*-mild-aGVHD recipients (Figure 12, B–E). Compared with TCD-BM-no-aGVHD recipients, WT-aGVHD recipients showed typical histopathology of aGVHD, including (a) lymphocytic infiltration, epithelial cell apoptosis, and crypt dropout; (b) epidermal hyperplasia, expansion of dermis, and loss of subcutaneous fat; (c) lymphocytic infiltration in hepatic portal triads; and (d) lymphocytic bronchiolitis. *Gzmk^–/–^*-mild-aGVHD recipients showed less tissue damage on day 14 after hematopoietic cell transplantation (HCT) (Figure 13A). As compared with control TCD-BM-no-aGVHD recipients, WT-aGVHD recipients had a marked increase of GZMK^+^CD8^+^ TRM cell infiltration in the intestines by multiplex immunofluorescence analysis. In contrast, almost no GZMK^+^CD8^+^ TRM cell infiltration in the intestines was observed in the *Gzmk^–/–^*-mild-aGVHD recipients (Figure 14A). Our data indicate that donor-derived GZMK^+^CD8^+^ TRM cell deficiency attenuates intestinal aGVHD severity.

## Discussion

In this study, we used scRNA-seq, scTCR-seq, and mIHC to investigate the differentiation trajectory, lineage commitment, clonal expansion, and functional properties of distinct CD8^+^ TRM cell subsets in human intestinal aGVHD. First, we provide a framework for better understanding the CD8^+^ TRM subset landscape. We identified a predominant subset of CD8^+^ TRM cells, the GZMK^+^CD8^+^ TRM subset. Second, we provided additional insights into the functions of this GZMK^+^CD8^+^ TRM subset, which exhibited enhanced clonal expansion and IFN signaling pathway–associated proinflammatory signatures. Third, the GZMK^+^CD8^+^ TRM subset displayed lineage bifurcation differentiation properties and differentiated more into a CD8^+^ TRM_eff_ subset in intestinal aGVHD. Fourth, high GZMK^+^CD8^+^ TRM subset infiltration is correlated with human intestinal aGVHD severity and poor prognosis. Therefore, our data support an association between human intestinal aGVHD and infiltration of the intestine with GZMK^+^CD8^+^ T cells with TRM features.

The TRM cell phenotype and behavior in different tissues can substantially differ (51), and these differences allow for a greater ability to explore transcriptionally distinct subsets of TRM cells. A previous study demonstrated that human intestinal CD8^+^ TRM cells possess CD69^+^CD103^+^ TRM and CD69^+^CD103^–^ TRM subsets, which have distinct localizations and functions (38). In our study of human intestinal aGVHD, we identified 4 functionally distinct CD8^+^ TRM subsets, including GZMK^+^CD8^+^ TRM, CX3CR1^+^CD8^+^ TRM, TIGIT^+^CD8^+^ TRM, and CCL4^+^CD8^+^ TRM. These CD8^+^ TRM subsets were donor-derived, consistent with Kean’s study (24). Kean et al. demonstrated that donor T cells infiltrate target organs and rapidly exhibit protein and transcriptional TRM hallmarks within 8 days after allo-HSCT. These donor CD8^+^ TRM cells drive gastrointestinal aGVHD (24). Similar to stem-like CD8^+^ T cells (41, 52), TRM-like CD8^+^ T_recir-stem_ subset cells express high levels of stemness markers (*TCF7*, *LEF1*, *SELL*, and *CCR7*). We identified a CD8^+^ TRM subset known as a GZMK^+^CD8^+^ TRM subset, which represent recent progeny cells of TRM-like CD8^+^ T_recir-stem_ cells. However, the GZMK^+^CD8^+^ TRM subset appears to be distinct from the TRM-like CD8^+^ T_recir-stem_ subset. The GZMK^+^CD8^+^ TRM subset cells express lower levels of stemness markers (*TCF7*, *LEF1*, *SELL*, and *CCR7*) and are less quiescent than the TRM-like CD8^+^ T_recir-stem_ subset by clonal expansion. The GZMK^+^CD8^+^ TRM subset cells express certain levels of both effector (*CX3CR1*, *KLRG1*, *FGFBP2*, and *CCL4*) and exhausted (*TIGIT*, *CTLA4*, *HAVCR2*, and *TOX*) markers. The *GZMK* expression levels were maximal in the GZMK^+^CD8^+^ TRM subset and progressively decreased during the CX3CR1^+^ TRM_eff_ and TIGIT^+^ TRM_exh_ trajectories.

A previous mouse study revealed that GVHD is locally maintained in target tissues by resident progenitor-like T cells (which represent the tissue-resident TCF-1^+^ subpopulation), which have the ability to divide and replenish the effector T cell pool (53). Conversely, TCF-1–expressing cells, characterized by features of exhaustion and identified as “exhausted progenitor” (Texp) CD8^+^ T cells, have also been implicated in chronic viral infections and antitumor responses (41, 54). Moreover, although the differentiation trajectory of exhausted T cells has largely been thought to follow a linear path, an increasing number of studies have suggested a bifurcated path (44, 55, 56). Our data support a bifurcation model of CD8^+^ TRM differentiation during human intestinal aGVHD, with TRM-like CD8^+^ T_recir-stem_ subset cells transitioning through GZMK^+^CD8^+^ TRM cells to become either CX3CR1^+^ TRM_eff_ cells or TIGIT^+^ TRM_exh_ cells. Fate-tracing analyses revealed that GZMK^+^CD8^+^ TRM cells have a spreading ability across pseudotime trajectories and increased metrics of clonal expansion and intercluster transition. The GZMK^+^CD8^+^ TRM subset exhibited increased clonality, and 80% of the expanded GZMK^+^CD8^+^ TRM clones lacked a detectable clonally expanded TRM-like CD8^+^ T_recir-stem_ counterpart. Spatial analyses among different TRM subsets revealed a strong spatial correlation between GZMK^+^CD8^+^ TRM cells and CX3CR1^+^ TRM_eff_ or TIGIT^+^ TRM_exh_ cells. In addition, the GZMK^+^CD8^+^ TRM subset differentiated more into a CX3CR1^+^ TRM_eff_ subset in intestinal aGVHD. Differentiation fate decisions may partially be influenced by environmental and inflammatory cues that are propagated during intestinal aGVHD, TCR avidity, and increased TCR signal strength. It has been demonstrated that increased avidity allows for increased numbers of durable TCR-pMHC interactions and leads to increased levels of TCR signaling downstream of ligand binding (44). Our data revealed that the signaling lymphocytic activation molecule family receptor (*SLAMF7*) was more highly transcribed and expressed by GZMK^+^CD8^+^ TRM cells, thereby potentially facilitating their recognition of antigens. Furthermore, TCR signaling module scores were highest in the GZMK^+^CD8^+^ TRM cells. These findings suggest that GZMK^+^CD8^+^ TRM cells may lose quiescent features upon TCR stimulation and undergo clonal expansion.

Jonsson et al. showed that GZMK^+^CD8^+^ T cells form a core population of tissue-associated T cells across diseases and human tissues (57). Mogilenko et al. identified GZMK^+^CD8^+^ T cells as a hallmark of immune aging and age-associated GZMK^+^CD8^+^ T cells could act as potential targets to address age-associated dysfunctions of the immune system (58). Gao et al. further identified circulating GZMK^+^CD8^+^ T cells as a hallmark of the pathogenesis of chronic GVHD–induced bronchiolitis obliterans syndrome (59). Our study revealed that the numbers and ratios of GZMK^+^CD8^+^ TRM subset infiltration were high in intestinal aGVHD patients. Higher GZMK^+^CD8^+^ TRM subset infiltration was associated with worse OS and DFS. However, CX3CR1^+^ TRM_eff_ and TIGIT^+^ TRM_exh_ infiltration was not associated with survival and prognosis. It has been demonstrated that proinflammatory effects are modulated by the activation of STAT1/IRF1 signaling (60–62). In addition, the STAT1-IRF1 complex can affect the antitumor and immunoregulatory actions of IFNs through IFN-stimulated genes (ISGs) (63–65). In our study, GZMK^+^CD8^+^ TRM cells from aGVHD patients expressed increased levels of STAT1 and IRF1, leading to increased transcription of ISGs, suggestive of priming mechanisms that augment IFN responses. Furthermore, GZMK^+^CD8^+^ TRM cells from aGVHD patients secreted more IFN-γ compared with patients without aGVHD and HCs. IFN-associated inflammatory programs may contribute to the intestinal immune microenvironment in aGVHD.

Our study has some limitations. Our findings were based on the GVHD prophylaxis regimen without posttransplant cyclophosphamide (PTCy). It has been demonstrated that PTCy significantly delayed T cell reconstitution and affected the T cell subsets by increasing regulatory T cells (Treg) while reducing naive CD4^+^ T cells in the early posttransplant period (66, 67). However, the reconstitution of CD8^+^ T cells showed a similar trend between PTCy and non-PTCY groups (66, 68). And CD8^+^ T cells primarily included effector memory cells (CD45RA^–^CCR7^–^) and terminally differentiated cells (CD45RA^+^CCR7^–^) in patients undergoing transplantation with PTCy (69). Nevertheless, a previous study further demonstrated that the global TCRβ repertoires of CD8^+^ T cells infiltrating the gastrointestinal tract had limited overlap with the repertoires found in the blood (69). Whether PTCy-based versus non–PTCy-based regimens have comparable GZMK^+^CD8^+^ TRM cell infiltration in the intestinal aGVHD patients is also worthy of validation in further study.

Our study highlights an important association of the GZMK^+^CD8^+^ TRM cell subset with human intestinal aGVHD and provides a rationale for future studies exploring whether this subset could be therapeutically targeted.

## Methods

### Sex as a biological variable.

For the human study, both male and female participants were included. Sex was considered as a biological variable in the clinical analyses. Sex-stratified analyses of GZMK^+^CD8^+^ TRM cells were not performed. For the animal study, only male mice were used to maintain experimental consistency.

### Patient population and clinical data.

Intestinal biopsy samples were obtained from 51 patients with intestinal aGVHD, 16 patients with no aGVHD, and 12 HCs (age: 29–66 years). All of these included patients who did not have severe active infections at the time of intestinal biopsy sample collection. aGVHD patients were diagnosed according to the National Comprehensive Cancer Network (NCCN) guidelines and graded based on modified Glucksberg criteria (26).

### Sample collection, staining, and single-cell sequencing.

Intestinal biopsy samples were obtained by colonoscopy and were either fixed in formalin and embedded in paraffin for histopathological and mIHC analyses or processed for scRNA-seq. Peripheral blood samples were collected for matched scRNA-seq analysis. CD8^+^ T cells from intestinal and peripheral blood suspensions were isolated by magnetic bead separation. H&E staining, mIHC, combined immunophenotyping with FISH-XY, scRNA-seq, and single-cell V(D)J sequencing were performed as described in the Supplemental Methods. Antibodies are listed in Supplemental Table 5. H&E-stained and immunostained sections were performed by investigators blinded to the study groups.

### scRNA-seq and V(D)J sequencing.

scRNA-seq and scTCR-seq data were processed using Cell Ranger (https://www.10xgenomics.com/support/software/cell-ranger) and analyzed with Seurat (https://satijalab.org/seurat/). Downstream analyses included clustering, donor/recipient assignment, differential gene expression analysis, pathway enrichment analysis, tissue residency scoring, pseudotime trajectory analysis, TCR clonotype analysis, and transcription factor/regulon analysis. Detailed filtering parameters, software versions, gene set scoring methods, trajectory inference, TCR analysis, and transcription factor analysis are provided in the Supplemental Methods.

### Mice.

C57BL/6 (H-2^b^) and BALB/c (H-2^d^) mice were obtained from the Animal Experiment Center of Southern Medical University, and the *Gzmk^–/–^* C57BL/6 (H-2^b^) mice were purchased from Shanghai Model Organisms Center, Inc. Mice were maintained in a specific pathogen–free room at the Animal Institute of Southern Medical University. Male, 8- to 12-week-old mice were used for animal experiments. All animal protocols were approved by the Southern Medical University Institutional Animal Care and Use Committee. BALB/c mice were irradiated at a dose of 850 cGy 8–10 hours before transplantation. Control recipients were injected with 2.5 × 10^6^ TCD-BM from WT C57BL/6 donors alone. aGVHD recipients were injected with 2.5 × 10^6^ TCD-BM (WT C57BL/6) together with sorted CD4^+^ T cells (0.25 × 10^6^) from WT C57BL/6 donor spleen and sorted CD8^+^ T cells (2.5 × 10^6^) from WT C57BL/6 or *Gzmk^–/–^* C57BL/6 donor spleen. The assessment and scoring of GVHD clinical symptoms and target organs were performed by investigators blinded to the experimental groups according to previous publications (70–72).

### Statistics.

Statistical tests for H&E staining and mIHC data were performed via GraphPad Prism 8, and *P* values were calculated using 2-tailed unpaired Student’s *t* tests. The results are presented as medians with 95% CIs. Multiple groups were compared in a pairwise manner using the Wilcoxon test. DEG analysis was performed with cutoffs of a Benjamini-Hochberg–adjusted *P* value of less than 0.05 and absolute (avg_log_2_FC) greater than 0.25. For binary logistic regression analyses, odds ratios (ORs) and 95% CIs were estimated. For Cox proportional hazards regression analyses, hazard ratios (HRs) and 95% CIs were estimated. Survival analysis was performed using the Kaplan-Meier method and tested with the log-rank test (Mantel-Cox). OS was defined as the time from the date of biopsy until death from any cause. DFS was defined as the time from the date of biopsy until disease relapse or death from any cause. A *P* value of less than 0.05 was considered significant.

### Study approval.

This study was approved by the ethics committee review board at Nanfang Hospital, Southern Medical University, and written informed consent was obtained from all of the healthy volunteers and patients in accordance with the Declaration of Helsinki before the initiation of the study.

### Data availability.

Values for all data points in graphs are reported in the Supporting Data Values file. scRNA-seq and scTCR-seq data in this study have been deposited in the NCBI Gene Expression Omnibus (GEO GSE284015 and GSE318630). Any additional information that supports the findings of this study is available from the corresponding author upon reasonable request.

## Author contributions

HJ and QL designed and supervised the entire study. HJ, YS, and YX wrote the manuscript. YS and YX performed and interpreted all the pathological and mIHC experiments, with substantial contributions from XL. CS performed animal experiments. The single-cell experiments and the acquisition and analysis of scRNA-seq and scTCR-seq data were carried out by YS and RS. ZF, RL, FH, and NX were primarily responsible for the collection and processing of peripheral blood and intestinal samples. LX, MD, and JS contributed to the analyses and interpretation of the clinical data. All authors read and approved the final manuscript.

## Conflict of interest

The authors have declared that no conflict of interest exists.

## Funding support

The following organizations provided financial support:

- Science and Technology Planning Project of Guangdong Province (no. 2024A0505090015).
- National Natural Science Foundation of China (no. 82570271).
- Noncommunicable Chronic Diseases–National Science and Technology Major Project (no. 2023ZD0501200).
- Clinical Research Project of Nanfang Hospital, Southern Medical University (no. 2023CR007).
- Science and Technology Project of Ganzhou City (no. GZ2024YLJ033).

## Supplementary Material

Supplemental data

Supplemental tables 2-5

Supporting data values

## Figures and Tables

**Table 1:**
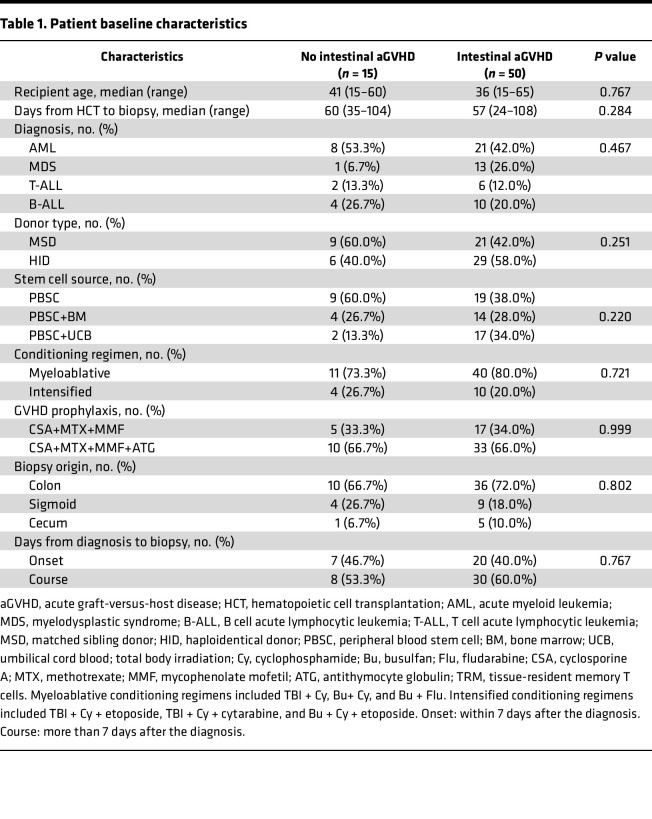
Patient baseline characteristics

**Table 2:**
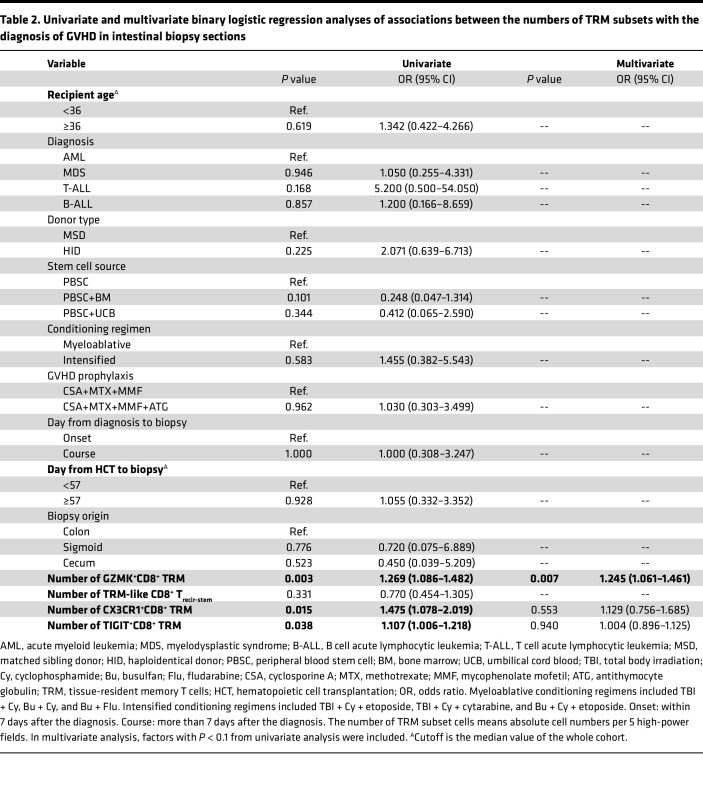
Univariate and multivariate binary logistic regression analyses of associations between the numbers of TRM subsets with the diagnosis of GVHD in intestinal biopsy sections

**Table 3:**
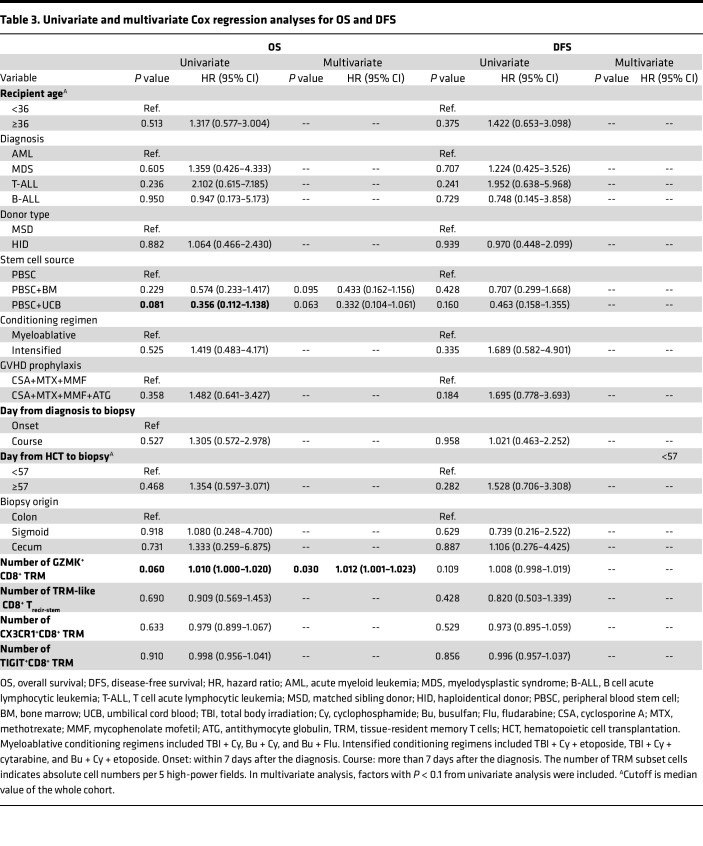
Univariate and multivariate Cox regression analyses for OS and DFS

## Version 1 07/22/2026

Electronic publication
