# Supplementary material for: GZMK-expressing tissue-resident memory CD8+ T cells participate in human intestinal acute graft-versus-host disease
Source: JCI Insight. 2026 Jul 22;11(14):e199156. doi: 10.1172/jci.insight.199156 (PMC13461161; doi:10.1172/jci.insight.199156)
Supplement: Supplementary file 1 [file jciinsight-11-199156-s235.pdf]

Methods:

Sample collection and processing

Intestinal biopsies were obtained via colonoscopy. Intestinal biopsy samples were fixed in formalin and embedded in paraffin for standard histopathological assessments or processed for scRNA-seq. Peripheral blood samples were obtained and processed for scRNA-seq. For scRNA-seq, samples were immediately subjected to single-cell dissociation within 3 hours after collection. Briefly, the tissue samples were first washed with Hanks Balanced Salt Solution without $Ca2^{+}$ and $Mg2^{+}$ (D-HBSS) (Thermo Fisher Scientific) to remove blood and fecal residues, after which they were enzymatically dissociated in digestion medium (1 mg/ml collagenase D (Roche), 100 µg/ml DNase I (Roche) in D-HBSS) for 1 hour. The digestion was terminated by adding 10 ml of washing buffer (2 mM EDTA, 2% FBS in D-HBSS). The cell suspension was filtered through a 30-µm nylon mesh (Miltenyi Biotec). Peripheral blood mononuclear cells (PBMCs) from peripheral blood samples were isolated using density gradient centrifugation by using lymphocyte separation medium (Biosharp). $CD8^{+}$ T cells from intestinal and PBMC suspensions were isolated by magnetic bead separation (human CD8 MicroBeads Kit, Miltenyi Biotec) according to the manufacturer's instructions. The numbers of $CD8^{+}$ T cells were determined using an automatic cell counter.

Hematoxylin and eosin (H&E) and multiplex immunohistochemistry

H&E and multiplex immunohistochemical staining was performed on 4-µm-thick sections. Pathological grade was evaluated according to the 4-tiered system on H&E-stained slides (1). A multicolor fluorescence kit based on tyramide signal amplification (TSA) was used following the manufacturer's protocol (abs50037, Absin Bioscience, China). The intestinal sections were baked, dewaxed, and rehydrated. Intestinal sections were subjected to antigen retrieval in Tris-EDTA

(pH=9.0) (Servicebio) and endogenous peroxidase blocking (Solarbio). Afterwards, the sections were blocked with blocking buffer (goat serum (Jackson), Fc receptor blocking solution (Biolegend) and glycine (Sigma) for 30 minutes and incubated with primary antibodies overnight at 4 ℃. Horseradish peroxidase (HRP)-labeled goat anti-rabbit/mouse secondary antibodies were used and developed with a fluorescent dye diluted with the signal amplification reagent that was provided with the kit (1:100). The sections were washed with antibody eluent to remove bound antibodies and subsequently incubated with another primary antibody. This process was repeated until all of the antigens were stained. Finally, DAPI was used to stain the nuclei. The antibodies used are listed in Supplemental Table 4. Slides were scanned via an LSM-980 microscope (Carl Zeiss) and a TissueFAXS Plus microscope (TissueGnostics) and analyzed using Imaris 10.1.0 and StrataQuest (TissueGnostics) software. In image analysis, spatial distance calculations were performed using MATLAB R2023a by randomly selecting fixed-size fields(20×) of view across different slices. For quantitative image analysis, the positive threshold for each marker fluorescence channel was determined based on nuclear or cytoplasmic staining patterns. Pathological evaluation of H&E-stained sections and visual assessment of immunostained sections were performed by investigators blinded to the study groups.

Combined immunophenotyping and FISH probe analysis

Intestinal sections were incubated with primary antibodies (anti-CD8 and anti-CD69) and HRP-labeled secondary antibodies before hybridization with the FISH probes. The sections were subsequently incubated with a fluorescent dye (TSA700 and TSA770) diluted with the signal amplification reagent. Afterwards, the nuclei were permeabilized with pepsin solution for 10 minutes at 37 ℃. The sections were sequentially dehydrated with 70%, 85%, and 100% alcohol and further processed via a FISH X/Y chromosome counting probe kit (Anbiping). After the FISH

probes were applied to the tissue areas, both the tissue and probes were codenatured at 85 ℃ for 5 minutes and incubated at 37 ℃ overnight, followed by posthybridization washing in 2×SSC/0.1% NP-40 at 37 ℃. The sections were counterstained with antifade medium with DAPI, which was provided with the kit. Images of the sections were acquired with a TissueFAXS Plus microscope. X/Y chromosomes were determined and recorded for $CD8^+CD69^+$ double-positive T cells.

Single-cell RNA and V(D)J sequencing

Single-cell RNA-seq and V(D)J-seq libraries were prepared using the SeekOne® DD Single Cell 5' Library Preparation Kit (SeekGene Catalog No. K00501) and the SeekOne® DD Single Cell V(D)J Enrichment Kit (SeekGene Catalog No. K00601), respectively, according to the manufacturer's instructions. The indexed sequencing libraries were cleaned using VAHTS DNA Clean Beads (Vazyme N411-03), analyzed via a Qubit Analyzer (Thermo Fisher Scientific Q33226) and Bio-Fragment Analyzer (Bioptic Qsep400), and sequenced via an Illumina NovaSeq 6000 with a PE150 read length.

scRNA-seq clustering leading to cell types

Unique molecular identifier (UMI) matrices for all of the samples were generated using Cell Ranger v5.0. The UMI data were imported into Seurat v5.0 (2) in R (4.4.0) for downstream analysis. Cells with more than 6,000 genes, fewer than 300 genes or more than 20% UMIs originating from mitochondrial genes were filtered and excluded. Genes that were expressed in fewer than 5 cells were also excluded. All of the samples were combined and clustered to filter out non-$CD8^+$ T cells from the datasets. Clusters with significant average expression of $CD8^+$ T cell genes were then extracted for further analysis. Standard data processing and normalization steps were performed using the Seurat NormalizeData and FindVariableFeatures.The number of counts, the fraction of mitochondrial genes, and the cell cycle scores were regressed out (S and G2M

scores were calculated by the CellCycleScoring function in Seurat). Afterwards, each patient dataset was further integrated to remove batch effects via CCA in the Seurat package. We excluded variable TCR (^TR.V) genes, variable Ig (^IG.V) genes, cell cycle genes (used for cell cycle scores) and mitochondrial genes (^MT-) from integration features used for downstream analysis. Analyses of the first 20 principal components of the integrated expression data were performed, followed by clustering with the FindNeighbors and FindClusters functions (Seurat). The RunUMAP function was used to visualize the datasets. Outlier and low-quality clusters were removed. Doublets were predicted using scDblFinder (3). Clusters in the resulting two-dimensional uniform manifold approximation and projection (UMAP) representation consisted of distinct cell types, which were identified based on the expression of marker genes. Annotations of donor- and recipient-derived cells were performed by deconvolution of the samples by using souporcell (4) on the aligned bam file produced via CellRanger. The $CD8^+$TRM cells that were defined by souporcell were manually labeled as either donors or recipients based on sex genotyping (x-linked gene: XIST and Y-linked gene: RPS4Y1).

Gene expression and pathway analysis

Differentially expressed genes (DEGs) were identified using the functions FindAllMarkers or FindMarkers (Seurat) for pairwise comparisons by using logfc.threshold=0.01, min.pct=0.1, and test.use=”wilcox”. Genes with Benjamini-adjusted *P* values of less than 0.05 and an average log-fold change of more than 0.25 were considered for analysis. KEGG, HALLMARK and GO pathway analyses of DEGs were performed via Gene Set Enrichment Analysis (GSEA) (5), with all of the expressed genes being utilized as the background gene list via the clusterProfiler (6) GSEA function. Only pathway gene sets with a Q value<0.05 and genes with a normalized enrichment score (NES) >0.5 or <0.5 were used. Gene set enrichment scores were calculated using

the AUCell algorithm (7). The resident, circulatory, stemness, effector and exhausted gene signatures were generated from previously reported studies (8-12). Immunologic signature gene sets were obtained from MSigDB (www.gsea-msigdb.org). The average gene expression was calculated using the AggregateExpression function (Seurat). Density plots of features were visualized using the Nebulosa package. The bubble plots, volcano plots , boxplots Venn diagrams and heatmaps were generated by using the ggplot2 package. A DEGs Circos plot was generated by using code (github.com/ayshwaryas/Tim3_Microglia _papercode).

Trajectory analysis

Pseudotime trajectories were computationally imputed in $CD8^+$TRM cells via the Slingshot (13) package. The TRM-like $CD8^+T_{recir\text{-}stem}$ cluster was considered as a root statistic when calculating the trajectories and the pseudotime value, followed by two-dimensional visualization on UMAP. The TradeSeq package was used to calculate gene expression and differences along the trajectories. Heatmaps were generated with the ComplexHeatmap (14) package. For Monocle3 (15) analysis, the Monocle tree structure was generated using the DDRTree algorithm with the top 75 differentially expressed genes from each Seurat cluster. For PAGA (16) analysis, a Python environment was established. The adjacency relationships and trajectories among the $CD8^+$TRM subsets were obtained via the RunPAGA and PAGAPlot functions in the SCP package.

Single-cell TCR-seq clonotype analysis

TCR alignment and annotation were performed with CellRanger v5.0. The package scRepertoire (17) was used for downstream analysis. TCRα- and β-chain complementarity-determining region 3 (CDR3) nucleotide sequences were combined for scRNA-seq. A total of 7,967 of 9,990 $CD8^+$TRM cells had paired full-length α and β chains that were sequenced. Cells missing an identifier were considered to be not observed (rather than not clonal). None of these TCR

sequences were shared between the patients. TCR clonotypes were defined as TCRs with the same CDR3 nucleotide sequences and VDJC gene sequence. Clone cells were defined as ≥ 2 cells with the same TCR sequence and binned into different categories (2-3 and 3+ cells). The cells of the TCR clonotypes from each patient were grouped for differential analysis and visualized. A bootstrap downsampling approach (100 iterations) was employed to normalize clonal expansion analysis across subsets of varying sizes, wherein 200 cells were randomly sampled with replacement from each of the four core subsets per iteration. TCR richness, which was defined as the number of unique TCRs divided by the total number of cells with a unique TCR, was calculated to assess clonotype diversity in the clusters and trajectories. Shared TCR clones were also calculated via the clonalOverlap function (scRepertoire), and the chord diagram was visualized via the Circlize package. A TCR Circos plot was generated by using code (github.com/aislyn/TCR_circos). The UpSet plot was generated via the UpSetR (18) package. The Transition Index was calculated via STARTRAC package (19).

Transcription factor analysis

The active transcription factor (TF) modules in $CD8^+$TRM cells were detected via the SCENIC[67] package. The normalized single-cell gene expression matrix was first filtered to exclude all of the genes that were detected in fewer than 15 cells. The RcisTarget database, which contains TF motif scores for gene promoters and around transcription start sites for the hg19 human reference genome, was downloaded from a specific network (resources.aertslab.org). The expression matrix was further filtered to include only genes that were available in the RcisTarget database. The remaining genes were used to compute a gene-gene correlation matrix for coexpression module detection using the random forest-based GENIE3 algorithm; additionally, the SCENIC package was used to perform TF network analysis to detect coexpression modules that were enriched with target genes

of each candidate TF from the RcisTarget database. The AUCell algorithm was used to compute a score for each TF module in each individual cell. Differential activity scores of the TFs in $CD8^+$TRM subsets were compared. The regulon specificity score (RSS) of the TRM subsets was calculated via the calcRSS function. Regulon modules were identified based on the Connection Specificity Index (CSI) (20), which is a context-dependent measure for identifying specific associated partners. The CSI indices were calculated using the scFunctions package.

Induction and assessment of murine GVHD

BALB/c mice were irradiated at a dose of 850 cGy 8-10 hours before transplantation. Control recipients were injected with 2.5×10^6 T-cell–depleted bone marrow cells (TCD-BM) from wild type (WT) C57BL/6 donors alone. Acute GVHD recipients were injected with 2.5×10^6 TCD-BM (WT C57BL/6) together with sorted $CD4^+$ T cells (0.25×10^6) from WT C57BL/6 donor spleen and sorted $CD8^+$ T cells (2.5×10^6) from WT C57BL/6 or $GZMK^{-/-}$ C57BL/6 donor spleen. T cell depletion from bone marrow was accomplished by using anti-CD4 and anti-CD8 Microbeads (Miltenyi Biotec, Germany), followed by passage through a MACS Column-based cell separation device (Miltenyi Biotec, Germany). Enrichment of $CD4^+$ T and $CD8^+$ T cells from spleen was accomplished by using mouse anti-CD4 and anti-CD8 Microbeads (Miltenyi Biotec, Germany). The purity of enrichment was >97%, whereas the purity after depletion was >99%.

References

1. Westerhoff M., Lamps L.W. Mucosal Biopsy After Bone Marrow Transplantation. *Surg Pathol Clin,* 2017; 10(4): 909-930.
2. Hao Y., Stuart T., Kowalski M.H., et al. Dictionary learning for integrative, multimodal and scalable single-cell analysis. *Nat Biotechnol,* 2024; 42(2): 293-304.
3. Germain P.L., Lun A., Garcia Meixide C., et al. Doublet identification in single-cell sequencing data using scDblFinder. *F1000Res,* 2021; 10(979.
4. Heaton H., Talman A.M., Knights A., et al. Souporcell: robust clustering of single-cell RNA-seq data by genotype without reference genotypes. *Nat Methods,* 2020; 17(6): 615-620.
5. Subramanian A., Tamayo P., Mootha V.K., et al. Gene set enrichment analysis: a knowledge-based approach for interpreting genome-wide expression profiles. *Proc Natl Acad Sci U S A,* 2005; 102(43): 15545-15550.
6. Wu T., Hu E., Xu S., et al. clusterProfiler 4.0: A universal enrichment tool for interpreting omics data.

Innovation (Camb), 2021; 2(3): 100141.

7. Aibar S., González-Blas C.B., Moerman T., et al. SCENIC: single-cell regulatory network inference and clustering. *Nat Methods,* 2017; 14(11): 1083-1086.
8. Milner J.J., Toma C., Yu B., et al. Runx3 programs CD8(+) T cell residency in non-lymphoid tissues and tumours. *Nature,* 2017; 552(7684): 253-257.
9. Mackay L.K., Rahimpour A., Ma J.Z., et al. The developmental pathway for CD103(+)CD8+ tissue-resident memory T cells of skin. *Nat Immunol,* 2013; 14(12): 1294-1301.
10. Anadon C.M., Yu X., Hänggi K., et al. Ovarian cancer immunogenicity is governed by a narrow subset of progenitor tissue-resident memory T cells. *Cancer Cell,* 2022; 40(5): 545-557.e513.
11. Li H., Van Der Leun A.M., Yofe I., et al. Dysfunctional CD8 T Cells Form a Proliferative, Dynamically Regulated Compartment within Human Melanoma. *Cell,* 2020; 181(3): 747.
12. Miller B.C., Sen D.R., Al Abosy R., et al. Subsets of exhausted CD8(+) T cells differentially mediate tumor control and respond to checkpoint blockade. *Nat Immunol,* 2019; 20(3): 326-336.
13. Street K., Risso D., Fletcher R.B., et al. Slingshot: cell lineage and pseudotime inference for single-cell transcriptomics. *BMC Genomics,* 2018; 19(1): 477.
14. Gu Z., Eils R., Schlesner M. Complex heatmaps reveal patterns and correlations in multidimensional genomic data. *Bioinformatics,* 2016; 32(18): 2847-2849.
15. Trapnell C., Cacchiarelli D., Grimsby J., et al. The dynamics and regulators of cell fate decisions are revealed by pseudotemporal ordering of single cells. *Nat Biotechnol,* 2014; 32(4): 381-386.
16. Wolf F.A., Hamey F.K., Plass M., et al. PAGA: graph abstraction reconciles clustering with trajectory inference through a topology preserving map of single cells. *Genome Biol,* 2019; 20(1): 59.
17. Borcherding N., Bormann N.L., Kraus G. scRepertoire: An R-based toolkit for single-cell immune receptor analysis. *F1000Res,* 2020; 9(47.
18. Conway J.R., Lex A., Gehlenborg N. UpSetR: an R package for the visualization of intersecting sets and their properties. *Bioinformatics,* 2017; 33(18): 2938-2940.
19. Zhang L., Yu X., Zheng L., et al. Lineage tracking reveals dynamic relationships of T cells in colorectal cancer. *Nature,* 2018; 564(7735): 268-272.
20. Fuxman Bass J.I., Diallo A., Nelson J., et al. Using networks to measure similarity between genes: association index selection. *Nat Methods,* 2013; 10(12): 1169-1176.

Supplemental Figures:

FigureS1

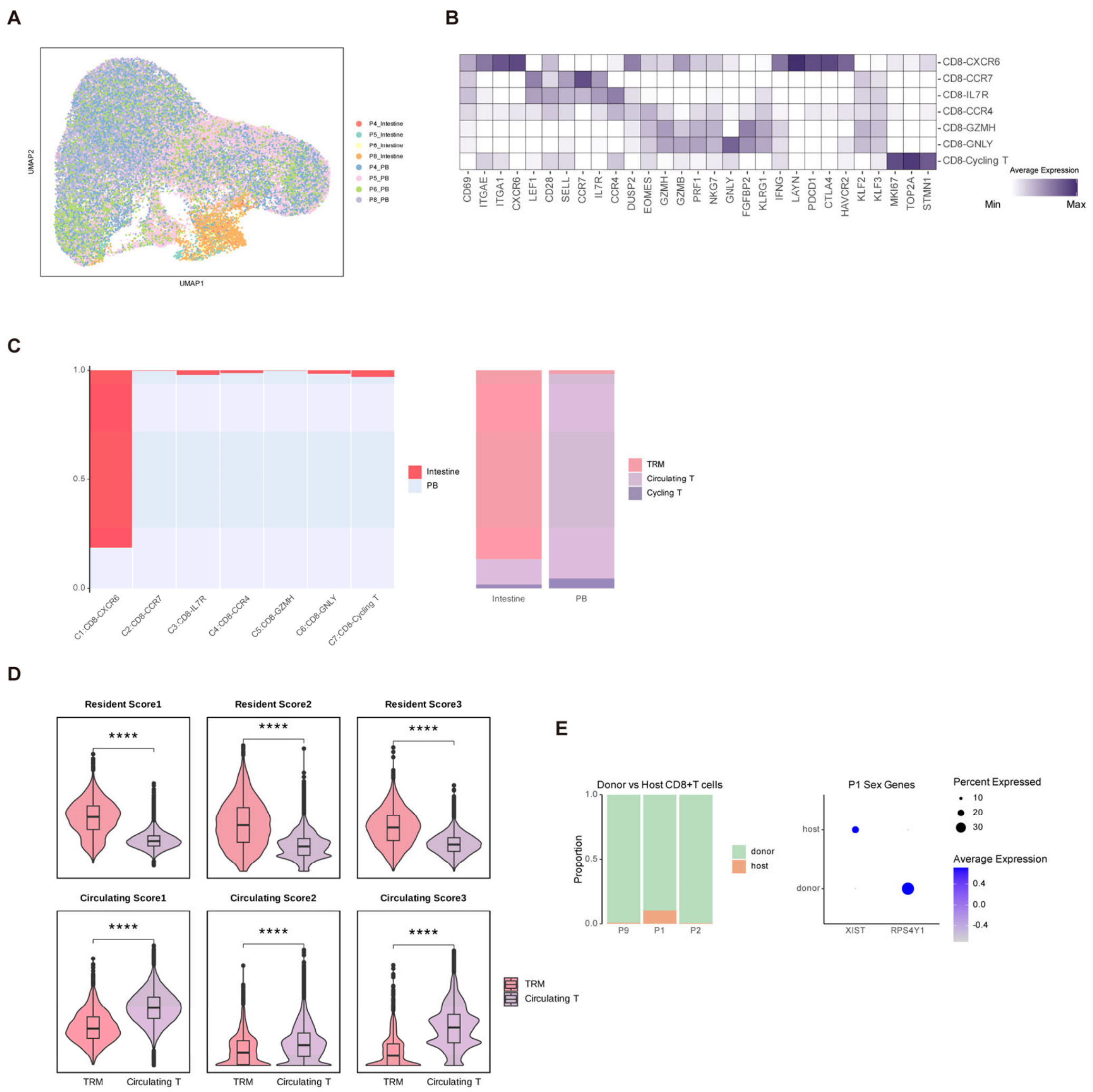

Fig. S1. Cell types and states detected via scRNA-seq analysis of CD8$^+$T cells from intestinal and PB samples.

(A) UMAP plot of CD8$^+$ T cells from 4 intestinal samples and 4 matched PB samples, colored according to each sample. **(B)** Heatmap displaying the average scaled expression levels of genes that were identified in the CD8$^+$ T cell subclusters. **(C)** Bar plots of the distribution of intestinal tissue and PB in each CD8$^+$ T cell cluster (Left). Bar plots of CD8$^+$ TRM cells, CD8$^+$ circulating T cells and Cycling T cells separated according to intestinal tissue and PB (Right). **(D)** Resident and circulating scores between CD8$^+$TRM cells and CD8$^+$ circulating T cells were calculated via the Aucell algorithm.****, $P < 0.0001$ according to Student's t test. **(E)** Bar plots showing host and donor cells in the intestinal samples of 3 intestinal aGVHD patients who received sex-

mismatched transplantations (Left). Dot plot showing sex genes (XIST and RPS4Y1) in a sample from Patient 5 (Right).

FigureS2

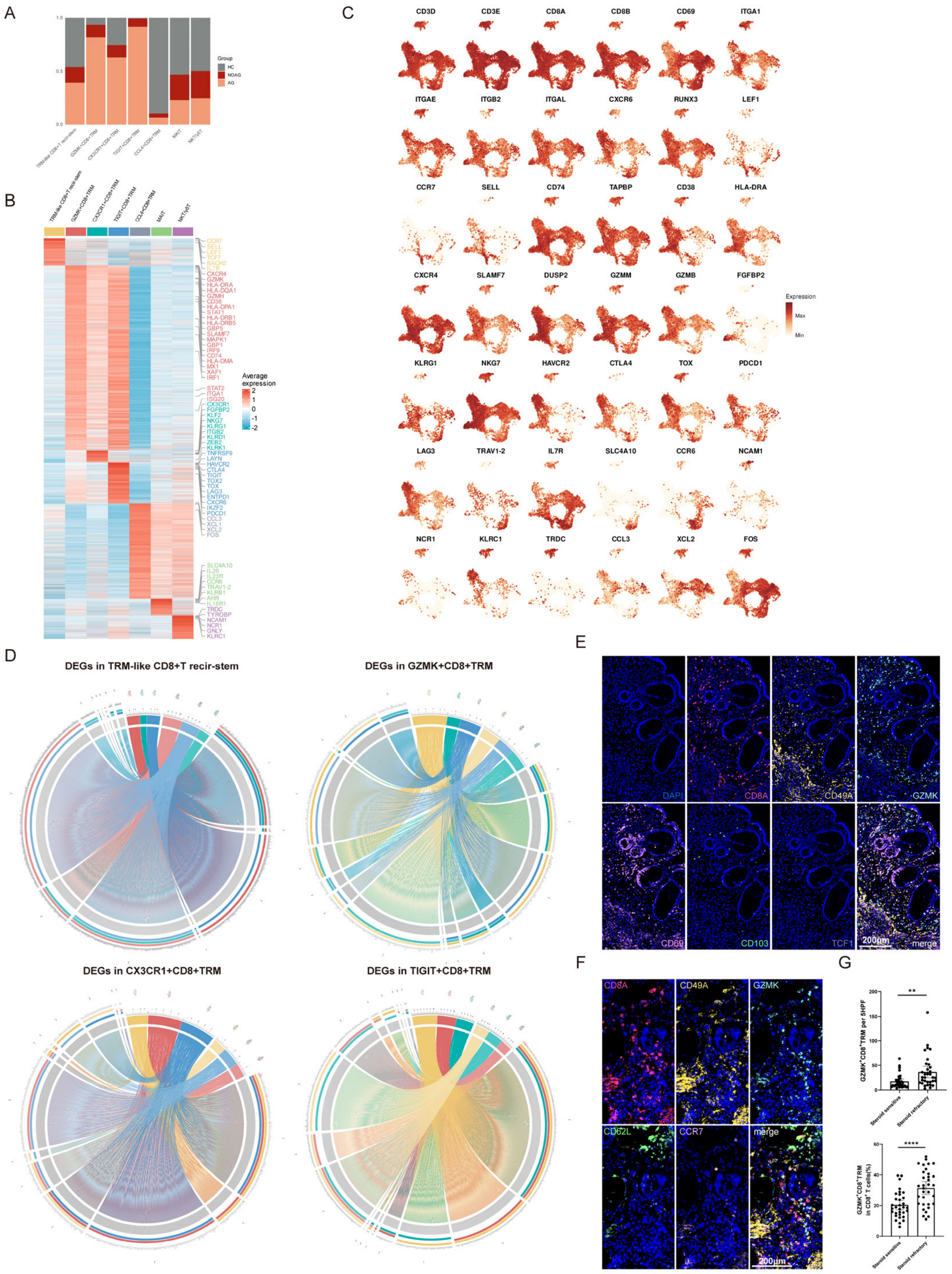

Fig. S2. Seven CD8$^+$ cell subsets were defined based on differential gene expression. **(A)** Stacked bar plots showing the distribution each CD8$^+$ cell subsets in HCs, no aGVHD and aGVHD groups. **(B)** Heatmap showing the average scaled expression levels of DEGs in each CD8$^+$ cell subsets. The representive genes were annotated. Benjamini-adjusted P values≤0.05 and

log2FC values≥0.25 were determined via the Wilcoxon rank-sum test. (**C**) Feature plots showing the expression of representive genes in $CD8^+$ cell subsets. (**D**) Circos plot comparison of up- and down-regulated DEGs among four $CD8^+$TRM cell subsets (TRM-like $CD8^+T_{recir\text{-}stem}$, $GZMK^+CD8^+$TRM, $CX3CR1^+CD8^+$TRM and $TIGIT^+CD8^+$TRM subsets). Only islands with DEGs shared by at least 2 of the 3 comparisons were included. Permutation test p-values were displayed as ***** $P \leq 0.00001$. (**E**) Representative images from intestinal samples subjected to multiplex immunohistochemical (mIHC) staining for CD69, CD103 and TCF1 in $GZMK^+CD8^+$ TRM cells. (**F**) Representative images from intestinal samples subjected to multiplex immunohistochemical (mIHC) staining for CD62L and CCR7 in $GZMK^+CD8^+$ TRM cells. (**G**) Percentages and numbers of the $GZMK^+CD8^+$TRM between steroid sensitive and steroid refractory groups**.** **, $P < 0.01$ and ****, $P < 0.0001$, according to Student's t test.

FigureS3

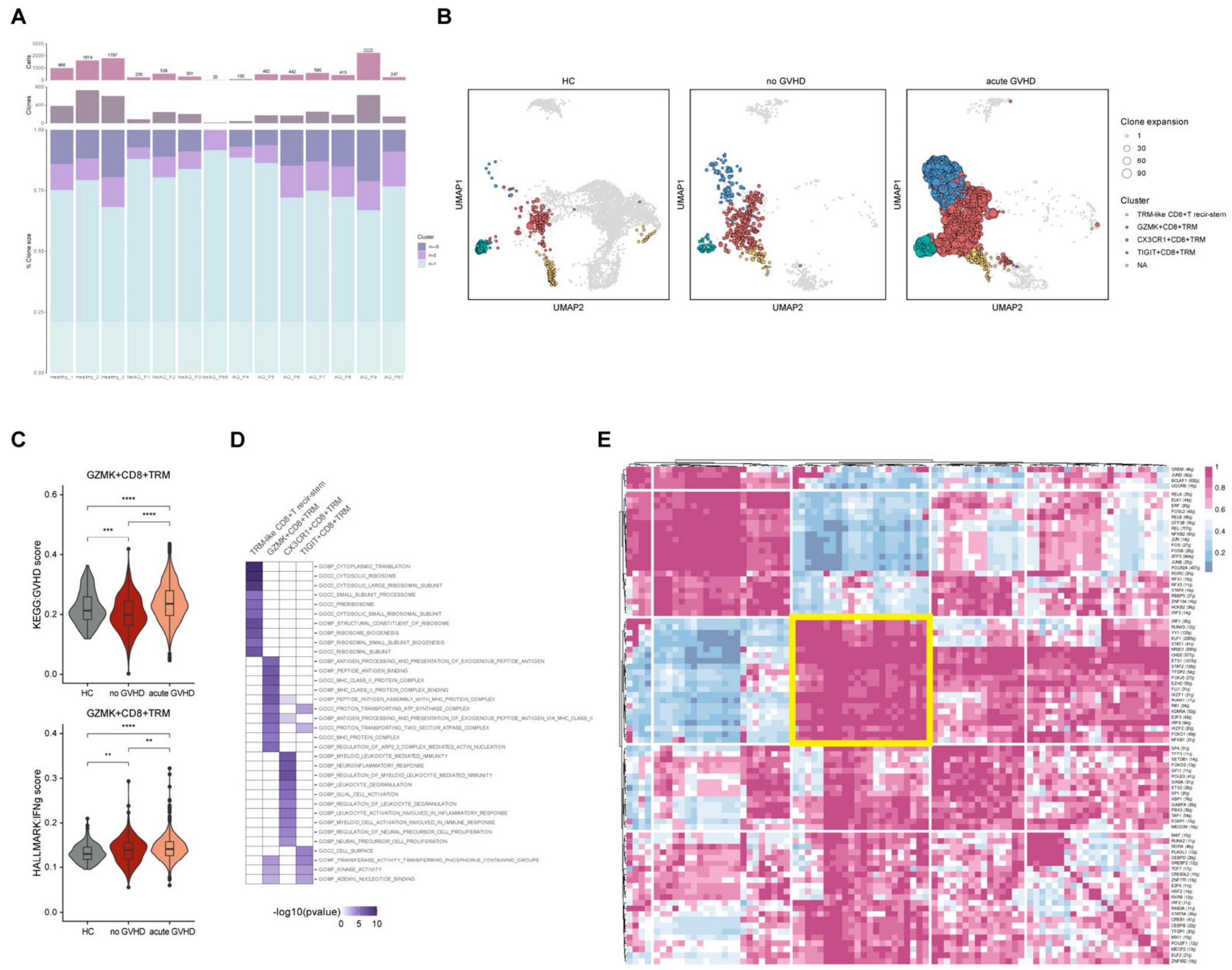

Fig. S3. Clonal expansion and genetic characteristics of $CD8^+$ TRM cell subset.

(A) Clonal composition of distinct $CD8^+$ TRM cell subsets. From top to bottom: the number of cells per individual sample, the number of distinct clones per individual sample, the distribution of clones by size (unique expansion (n=1), moderate expansion (n=2), and high expansion(n >=3)) showing the $CD8^+$ TRM cell subset composition of the clones of each sample, stratified according to clone size in individual samples. (**B)** UMAP embedding of the scRNA-seq data of T cells that were mapped to TCR clone information in HCs, no aGVHD and aGVHD groups, color-coded according to four $CD8^+$ TRM cell subsets (TRM-like $CD8^+T_{recir\text{-}stem}$, $GZMK^+CD8^+$TRM, $CX3CR1^+CD8^+$TRM and $TIGIT^+CD8^+$TRM). Clonal expansion, which was defined by a shared CDR3 nucleotide sequence and V and J gene usage, is depicted according to marker size. (**C)** Violin plot of enrichment GVHD and IFN-γ scores that were calculated for $GZMK^+CD8^+$TRM cell subsets among HCs, no aGVHD and aGVHD groups. (**D)** GSEA of enriched pathways (GO) for each $CD8^+$ TRM cell subset (q value<0.05). (**E)** Identified regulon modules based on the regulon Connection Specificity Index (CSI) matrix. The module included in the yellow box is associated with $GZMK^+CD8^+$ TRM cell subset.

FigureS4

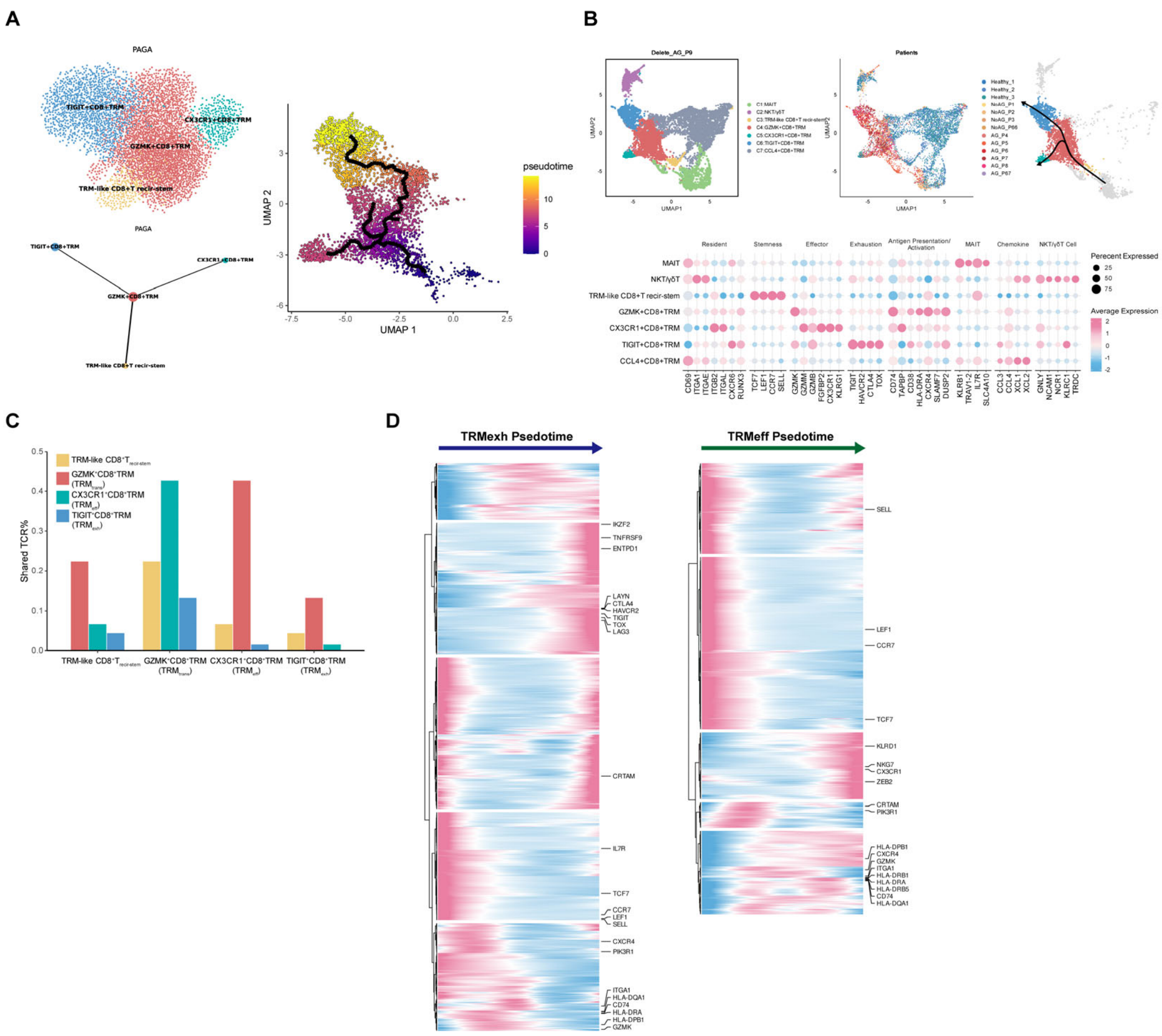

Fig. S4. Analysis of clonally expanding CD8$^+$ TRM cell subset trajectories.

(A) Differentiation trajectories of CD8$^+$ TRM cell subsets using PAGA and Monocle3 analysis. **(B)** After excluding the patient 09, UMAP plot, pseudotime trajectories and bubble plot of CD8$^+$ TRM cell subsets. **(C)** Bar plot showing the shared TCR weights for the CD8$^+$ TRM cell subsets. **(D)** Gene expression dynamics along the CD8$^+$ TRM$_{eff}$ trajectory and TRM$_{exh}$ trajectory. The genes are clustered into four gene sets, with each set characterized by specific expression profiles, as depicted by a selection of marker genes that are characteristic of each subset.

Supplemental Table 1 Clinical data for each biopsy sample.

Patient	Experiment content	Day after HSCT	Stages of intestinal acute GVHD	Grades of clinical acute GVHD	Origin	Age	Gender	Diagnosis	Donor type	Conditioning regimen	GVHD prophylaxis	Bx timepoint
Patient 01	mIHC+H&E+scRNAseq+scTCRseq	43	0	0	Colon	48	Female	B-ALL	MSD	Myeloablative	CSA+MTX+MMF+ATG	Onset
Patient 02	mIHC+H&E+scRNAseq+scTCRseq	84	0	0	Colon	41	Female	AML	HID	Myeloablative	CSA+MTX+MMF+ATG	Onset
Patient 03	mIHC+H&E+scRNAseq+scTCRseq	46	0	0	Colon	15	Female	B-ALL	HID	Myeloablative	CSA+MTX+MMF+ATG	Course
Patient 04	mIHC+H&E+scRNAseq+scTCRseq	78	1	2	Colon	32	Female	MDS	MSD	Myeloablative	CSA+MTX+MMF+ATG	Onset
Patient 05	mIHC+H&E+scRNAseq+scTCRseq	37	2	3	Sigmoid	34	Male	B-ALL	MSD	Intensified	CSA+MTX+MMF+ATG	Onset
Patient 06	mIHC+H&E+scRNAseq+scTCRseq	74	3	4	Colon	28	Female	MDS	HID	Myeloablative	CSA+MTX+MMF+ATG	Onset
Patient 07	mIHC+H&E+scRNAseq+scTCRseq	38	4	4	Colon	62	Male	AML	HID	Myeloablative	CSA+MTX+MMF+ATG	Onset
Patient 08	mIHC+H&E+scRNAseq+scTCRseq	87	4	4	Colon	22	Male	T-ALL	HID	Intensified	CSA+MTX+MMF+ATG	Course
Patient 09	mIHC+H&E+scRNAseq+scTCRseq	57	4	4	Colon	35	Male	MDS	MSD	Myeloablative	CSA+MTX+MMF+ATG	Course
Patient 10	mIHC+H&E	93	0	0	Sigmoid	29	Male	T-ALL	MSD	Intensified	CSA+MTX+MMF+ATG	Course
Patient 11	mIHC+H&E	56	0	1	Colon	59	Female	T-ALL	MSD	Intensified	CSA+MTX+MMF+ATG	Onset
Patient 12	mIHC+H&E	48	0	0	Sigmoid	48	Male	AML	MSD	Myeloablative	CSA+MTX+MMF+ATG	Course
Patient 13	mIHC+H&E	35	0	0	Colon	46	Female	B-ALL	HID	Myeloablative	CSA+MTX+MMF+ATG	Course
Patient 14	mIHC+H&E	61	0	0	Colon	34	Male	AML	MSD	Intensified	CSA+MTX+MMF	Onset
Patient 15	mIHC+H&E+FISH	47	0	0	Colon	26	Female	AML	MSD	Myeloablative	CSA+MTX+MMF	Course
Patient 16	mIHC+H&E+FISH	74	0	0	Colon	45	Female	AML	MSD	Myeloablative	CSA+MTX+MMF	Onset
Patient 17	mIHC+H&E	82	0	0	Sigmoid	60	Male	MDS	MSD	Myeloablative	CSA+MTX+MMF	Course
Patient 18	mIHC+H&E	84	0	0	Sigmoid	24	Male	AML	HID	Myeloablative	CSA+MTX+MMF+ATG	Course
Patient 19	mIHC+H&E	60	0	1	Cecum	16	Male	AML	HID	Myeloablative	CSA+MTX+MMF+ATG	Onset
Patient 20	mIHC+H&E	52	0	1	Colon	48	Female	AML	HID	Myeloablative	CSA+MTX+MMF+ATG	Onset
Patient 21	mIHC+H&E	85	0	0	Colon	30	Female	B-ALL	MSD	Intensified	CSA+MTX+MMF	Course
Patient 22	mIHC+H&E+FISH	77	1	2	Colon	39	Female	MDS	MSD	Myeloablative	CSA+MTX+MMF	Onset
Patient 23	mIHC+H&E+FISH	46	1	2	Colon	53	Female	B-ALL	HID	Myeloablative	CSA+MTX+MMF+ATG	Course
Patient 24	mIHC+H&E	58	1	2	Colon	29	Male	AML	HID	Myeloablative	CSA+MTX+MMF+ATG	Course
Patient 25	mIHC+H&E	92	1	2	Colon	15	Female	AML	HID	Myeloablative	CSA+MTX+MMF+ATG	Course
Patient 26	mIHC+H&E	62	1	2	Sigmoid	33	Male	AML	HID	Myeloablative	CSA+MTX+MMF+ATG	Course
Patient 27	mIHC+H&E	71	1	2	Sigmoid	36	Male	AML	HID	Myeloablative	CSA+MTX+MMF+ATG	Onset
Patient 28	mIHC+H&E	52	1	2	Cecum	46	Male	T-ALL	MSD	Intensified	CSA+MTX+MMF	Onset
Patient 29	mIHC+H&E	45	3	3	Colon	34	Male	B-ALL	MSD	Myeloablative	CSA+MTX+MMF	Course
Patient 30	mIHC+H&E+FISH	58	2	3	Colon	43	Female	MDS	MSD	Myeloablative	CSA+MTX+MMF	Course
Patient 31	mIHC+H&E	68	2	3	Cecum	58	Male	MDS	MSD	Myeloablative	CSA+MTX+MMF	Course
Patient 32	mIHC+H&E	75	2	3	Cecum	59	Male	MDS	HID	Myeloablative	CSA+MTX+MMF+ATG	Course
Patient 33	mIHC+H&E	27	3	3	Colon	25	Male	AML	HID	Myeloablative	CSA+MTX+MMF+ATG	Onset
Patient 34	mIHC+H&E+FISH	71	3	3	Cecum	26	Female	B-ALL	HID	Myeloablative	CSA+MTX+MMF+ATG	Course
Patient 35	mIHC+H&E	98	3	3	Colon	63	Female	AML	HID	Myeloablative	CSA+MTX+MMF+ATG	Course
Patient 36	mIHC+H&E	88	3	3	Colon	38	Male	MDS	MSD	Myeloablative	CSA+MTX+MMF	Course
Patient 37	mIHC+H&E	54	3	3	Colon	51	Male	AML	MSD	Myeloablative	CSA+MTX+MMF+ATG	Onset
Patient 38	mIHC+H&E+FISH	39	3	3	Sigmoid	56	Female	AML	HID	Myeloablative	CSA+MTX+MMF+ATG	Course
Patient 39	mIHC+H&E	61	4	4	Sigmoid	56	Male	MDS	MSD	Myeloablative	CSA+MTX+MMF	Onset
Patient 40	mIHC+H&E+FISH	69	4	3	Colon	40	Male	AML	MSD	Myeloablative	CSA+MTX+MMF	Onset
Patient 41	mIHC+H&E	25	4	4	Colon	34	Male	AML	HID	Myeloablative	CSA+MTX+MMF+ATG	Onset
Patient 42	mIHC+H&E	72	4	4	Colon	18	Male	B-ALL	HID	Intensified	CSA+MTX+MMF+ATG	Onset
Patient 43	mIHC+H&E	42	4	4	Colon	49	Male	MDS	HID	Intensified	CSA+MTX+MMF+ATG	Course
Patient 44	mIHC+H&E+FISH	57	4	3	Colon	27	Female	T-ALL	MSD	Intensified	CSA+MTX+MMF	Onset
Patient 45	mIHC+H&E	45	4	4	Colon	51	Male	AML	MSD	Intensified	CSA+MTX+MMF	Course
Patient 46	mIHC+H&E	88	4	4	Sigmoid	56	Male	AML	MSD	Myeloablative	CSA+MTX+MMF	Course
Patient 47	mIHC+H&E	77	4	4	Colon	44	Female	B-ALL	HID	Myeloablative	CSA+MTX+MMF+ATG	Course
Patient 48	mIHC+H&E	46	4	4	Colon	33	Male	T-ALL	HID	Intensified	CSA+MTX+MMF+ATG	Course
Patient 49	mIHC+H&E+FISH	50	4	4	Colon	39	Male	MDS	MSD	Myeloablative	CSA+MTX+MMF	Course
Patient 50	mIHC+H&E	34	4	4	Sigmoid	31	Male	B-ALL	HID	Myeloablative	CSA+MTX+MMF+ATG	Onset
Patient 51	mIHC+H&E+FISH	46	4	4	Sigmoid	37	Female	AML	HID	Myeloablative	CSA+MTX+MMF+ATG	Course
Patient 52	mIHC+H&E	47	4	4	Sigmoid	65	Male	AML	HID	Myeloablative	CSA+MTX+MMF+ATG	Onset
Patient 53	mIHC+H&E	80	4	4	Colon	29	Female	T-ALL	MSD	Intensified	CSA+MTX+MMF	Onset
Patient 54	mIHC+H&E	45	4	4	Colon	34	Female	AML	HID	Myeloablative	CSA+MTX+MMF+ATG	Onset
Patient 55	mIHC+H&E+FISH	68	4	4	Colon	54	Female	MDS	MSD	Myeloablative	CSA+MTX+MMF	Course
Patient 56	mIHC+H&E	54	4	4	Colon	55	Male	AML	MSD	Myeloablative	CSA+MTX+MMF	Course
Patient 57	mIHC+H&E	32	4	4	Colon	25	Male	MDS	MSD	Myeloablative	CSA+MTX+MMF	Onset
Patient 58	mIHC+H&E	61	4	4	Colon	23	Male	B-ALL	HID	Myeloablative	CSA+MTX+MMF+ATG	Course
Patient 59	mIHC+H&E+FISH	24	4	4	Cecum	46	Female	AML	MSD	Myeloablative	CSA+MTX+MMF	Onset
Patient 60	mIHC+H&E	45	4	4	Colon	35	Male	B-ALL	HID	Myeloablative	CSA+MTX+MMF+ATG	Course
Patient 61	mIHC+H&E	24	4	4	Colon	28	Female	T-ALL	HID	Intensified	CSA+MTX+MMF+ATG	Course
Patient 62	mIHC+H&E	31	4	4	Colon	35	Male	B-ALL	HID	Myeloablative	CSA+MTX+MMF+ATG	Course
Patient 63	mIHC+H&E	92	4	4	Colon	34	Male	AML	HID	Myeloablative	CSA+MTX+MMF+ATG	Course
Patient 64	mIHC+H&E	58	4	4	Colon	36	Female	AML	HID	Myeloablative	CSA+MTX+MMF+ATG	Course
Patient 65	mIHC+H&E	48	4	4	Colon	36	Male	AML	HID	Myeloablative	CSA+MTX+MMF+ATG	Course
Patient 66	scRNAseq+scTCRseq	69	0	0	Colon	75	Male	AML	HID	Myeloablative	CSA+MTX+MMF+ATG	Onset
Patient 67	scRNAseq+scTCRseq	44	4	4	Colon	22	Male	AML	HID	Myeloablative	CSA+MTX+MMF+ATG	Onset

Experiment content: mIHC: multiplex immunohistochemistry, H&E: hematoxylin and eosin, FISH: fluorescence in situ hybridization.
Diagnosis: AML: acute myeloid leukemia, B-ALL: B-cell acute lymphocytic leukemia, T-ALL: T-cell acute lymphocytic leukemia, MDS: myelodysplastic syndrome.
Donor type: MSD: matched sibling donor, HID: haploidentical donor.
Conditioning regimen: Myeloablative conditioning regimens include TBI (total body irradiation) + Cy (cyclophosphamide), Bu (busulfan)+Cy, and Bu + Flu (fludarabine). Intensified conditioning regimens include TBI + Cy + etoposide, TBI + Cy + cytarabine, and Bu + Cy + etoposide.
GVHD prophylaxis: CSA: cyclosporine A, MTX: methotrexate, MMF: mycophenolate mofetil, ATG: antithymocyte globulin.
Bx timepoint: Onset: within 7 days after the diagnosis; Course: more than 7 days after the diagnosis.
